# Gold Acyclic Diaminocarbene Complexes as Selective and Potent Agents for Multitarget Cancer Therapy

**DOI:** 10.1021/acs.inorgchem.5c00579

**Published:** 2025-05-05

**Authors:** María Gil-Moles, Melanie Aliaga-Lavrijsen, Sara Montanel-Pérez, Isabel Marzo, M. Dolores Villacampa, M. Concepción Gimeno

**Affiliations:** † Departamento de Química Inorgánica, Instituto de Síntesis Química y Catálisis Homogénea (ISQCH) CSIC-Universidad de Zaragoza, Zaragoza 50009, Spain; ‡ Departamento de Bioquímica y Biología Celular, Universidad de Zaragoza, Zaragoza 50009, Spain

## Abstract

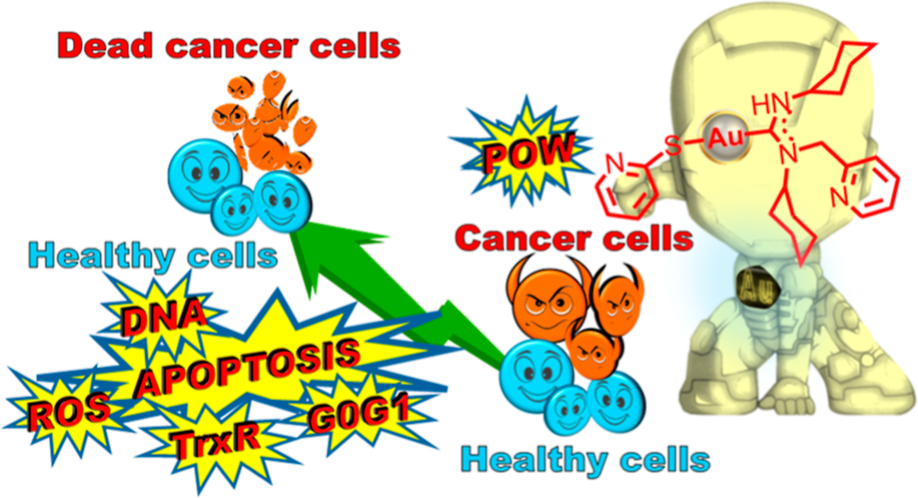

Gold acyclic diaminocarbene (ADC) complexes represent a promising, yet underexplored, class of chemotherapeutics. ADCs offer superior flexibility and stronger sigma donation compared with traditional N-heterocyclic carbenes, making them ideal ligands for stable gold-based drugs. In this study, a series of gold ADC complexes were synthesized via the nucleophilic addition of amines to [AuCl(CNCy)], yielding three structural families: gold-chloride-ADC (chiral and achiral), bis(carbene), and thiolate-gold-ADC complexes. Extensive characterization, including X-ray diffraction, revealed noncovalent interactions, such as hydrogen bonding and aurophilic contacts, that significantly shape their molecular architecture. These complexes exhibit potent cytotoxicity (IC_50_ in submicromolar) against drug-resistant cancer cell lines (A549, HCT116 WT, Jurkat, MiaPaca2), with some showing high selectivity toward cancer cells over healthy lymphocytes (selectivity index up to 74). Mechanistic investigations indicate that they disrupt mitochondrial function, elevate reactive oxygen species (ROS), and, in the case of bis(carbene) species, bind DNA. Apoptosis is induced at low concentrations, while higher doses trigger alternative death pathways. Notably, they also strongly inhibit thioredoxin reductase (TrxR), comparable in potency to auranofin. The combination of ROS induction, DNA interaction, mitochondrial disruption, and TrxR inhibition highlights the multitargeted anticancer potential of gold-ADC complexes and supports their further development as selective and effective chemotherapeutic agents.

## Abstract 2

Gold acyclic diaminocarbene (ADC) complexes represent a promising, yet underexplored, class of chemotherapeutics.

## Introduction

Metal-based drugs are widely employed in modern diagnosis and therapy, offering innovative solutions for challenging medical conditions.^1−4^ Compared with pure organic molecules, these compounds combine the unique characteristics of the metal ions with those of organic ligands, enabling the development of multifunctional therapeutic agents with enhanced potential. Often designed as prodrugs, metallodrugs are activated through mechanisms such as ligand substitution, redox reactions, or light activation.^5^ These properties together with the different activation pathways grant them unique properties and enhanced functionalities, particularly in combating cancer progression.^6,7^ Notably, one of their key advantages is their ability to function as multitargeting agents.^8,9^

Multitarget strategies in cancer therapy are designed to address the limitations of single-target approaches, which frequently result in drug resistance and diminished efficacy due to the intricate and adaptive nature of cancer. By simultaneously targeting multiple molecular pathways, cellular mechanisms, or tumor components, these strategies aim to deliver more effective therapeutic outcomes.

The ongoing search for new cancer therapies as alternatives to platinum-based drugs, due to their severe side effects and resistance issues, has driven interest in novel metal-based drugs with unique biological properties and targets. Gold derivatives have garnered attention, especially due to their ability to interact with multiple enzymes, highlighting their potential as multitarget agents.^10−13^

**Figure 1:**
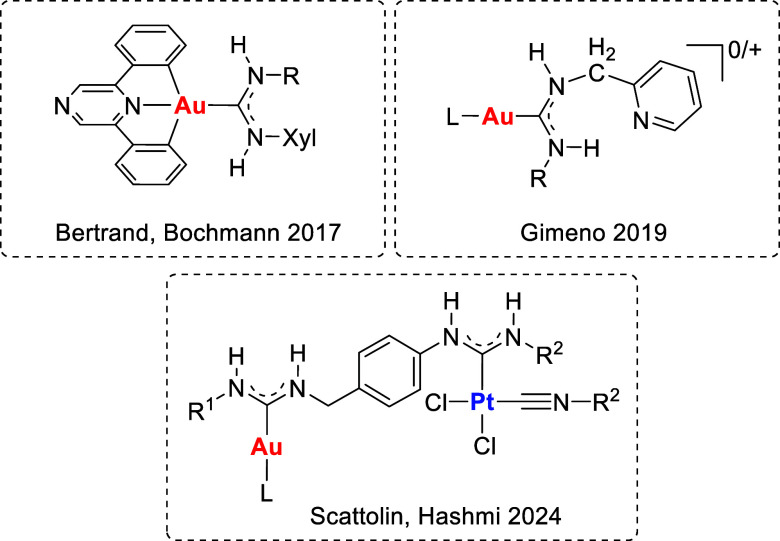
Gold ADC complexes with antitumor properties.

In the development of gold chemotherapeutics, ligand design plays a pivotal role in addressing key challenges such as stability, cellular uptake, and selectivity. In this sense, acyclic diaminocarbene (ADC) ligands represent a cutting-edge class of carbene ligands, distinguished by their open-chain, noncyclic structure featuring two nitrogen atoms bound to a central carbene carbon. Unlike the rigid cyclic framework of N-heterocyclic carbenes (NHCs), ADCs boast an adaptable structure that grants exceptional flexibility and opens up a wider range of geometries when bound to metal centers. This unique structural freedom, combined with their high electron density, makes ADCs an invaluable tool for catalysis, coordination chemistry, and material chemistry.^14,15^ While ADC gold complexes have well-established applications in catalysis^16−21^ and materials science,^22,23^ their potential in biological systems remains largely unexplored. However, N-heterocyclic gold(I) carbenes have been extensively studied in medicine, particularly as antitumor agents, with several showing promising efficacy.^24,25^ By comparison, there are few reports on the antitumor activity of gold(I) and gold(III) acyclic diaminocarbene derivatives. To date, research has been limited to a few reports, including gold(I) and gold(III) complexes from our group,^26,27^ those reported by Bertrand, Bochmann, and co-workers,^28,29^ and the bimetallic gold–platinum species recently reported by Scattolin, Hashmi, and co-workers (Figure 1).^30^ A wide variation in cytotoxicity has been observed among these derivatives, with IC_50_ values ranging from the high micromolar to the nanomolar range. Furthermore, their mechanisms of action are poorly understood. To date, the evaluated complexes have shown moderate inhibition of TrxR and low ROS production. However, other potential biological targets have not yet been investigated. This fact highlights that further investigation of the mechanism and improved selectivity toward tumor cells are still needed.

To address this gap, this study centers on the synthesis of a series of chiral and achiral gold acyclic diaminocarbene complexes with diverse stoichiometries. Specifically, three families of complexes will be evaluated: gold-chloride-ADC, bis(carbene), and thiolate-gold-ADC complexes in order to determine the influence of the auxiliary ligands in the anticancer properties as well as in stability. The cytotoxic properties of these complexes have been rigorously evaluated across multiple cancer cell lines as well as in healthy lymphocytes. Some of the complexes show promising activity and selectivity, and their mechanisms of action have been thoroughly investigated, revealing new insights into their potential for targeted cancer therapy.

## Results and Discussion

### Synthesis and Characterization of Gold Complexes

One of the most commonly used methods for synthesizing metal ADC complexes is the nucleophilic attack of amines on isocyanide metal derivatives,^31,32^ although other synthetic procedures have been reported.^33^ This approach is especially effective for gold isocyanide complexes, which typically exhibit a strong electrophilic character at the carbon atom of the isocyanide group, facilitating the straightforward synthesis of acyclic diaminocarbene complexes through reactions with amines.^34^

**Scheme 1:**
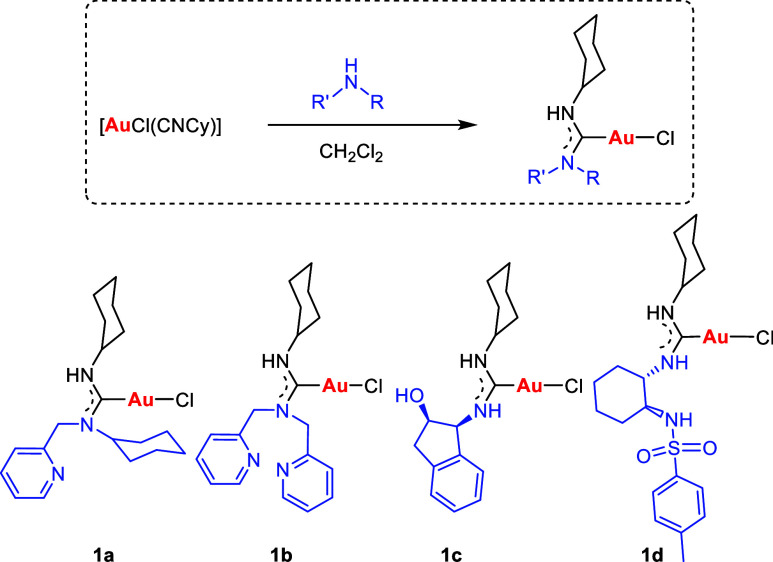
Synthesis of Gold(I) Acyclic Diaminocarbenes **1**

In our investigations, we have reacted the isocyanide complex [AuCl(CNCy)] with various amines, including those featuring pyridine substituents and chiral structures. A nucleophilic attack occurs when the NH group of secondary amines, such as *N*-(pyridylmethyl)cyclohexylamine and di(pyridylmethyl)amine, targets the electron-deficient carbon of the isocyanide, which serves as an electrophilic center, thus leading to the formation of the corresponding amino carbene. Furthermore, we have explored gold complexes with chiral amine and diamine scaffolds, including aminoindanol and 1,2-cyclohexanediamine, as illustrated in Scheme 1.

All complexes were characterized by ^1^H and ^13^C{^1^H}-APT NMR spectroscopy (see Supporting Information Figures S1–S8), IR spectroscopy, and mass spectrometry. For all of the complexes, the infrared (IR) spectra showed key absorptions, including the prominent ν(Au–Cl) band at approximately 325 cm^–1^, the ν(C=N) band of the newly formed carbenes at 1587 cm^–1^, and the NH band around 2932 cm^–1^. The mass spectra exhibited molecular ion peaks corresponding to protonated or sodium-cationic species [M + H]^+^ or [M + Na]^+^ or fragments arising at the loss of the chloride ligand [M – Cl]^+^. The ^1^H NMR spectra for complexes **1a–c** displayed the expected signals without any indication of rotamers. However, for complex **1d**, three distinct rotamers were clearly observed in the signals of the tolyl group. These resonances were simplified upon recording the spectrum in DMSO-*d*_6_ at elevated temperatures, up to 394 K (see Supporting Information, Figure S9). In the ^13^C APT NMR spectra, all signals could be distinctly assigned, including those corresponding to the cyclohexyl moieties. Notably, the resonances for the carbene carbon appeared at 192.8 and 193.0 ppm for complexes **1a** and **1b**, respectively. These positions are deshielded compared to those in [AuX(NHC)] (X = Cl, Br) complexes with both aryl and alkyl substituents (range: 172–174 ppm). This deshielding may be attributed to reduced electron density on the carbene carbon, likely caused by stronger metal–ligand interactions, lower donor capacity of the ligand, or other contributing factors.^35,36^

**Scheme 2:**
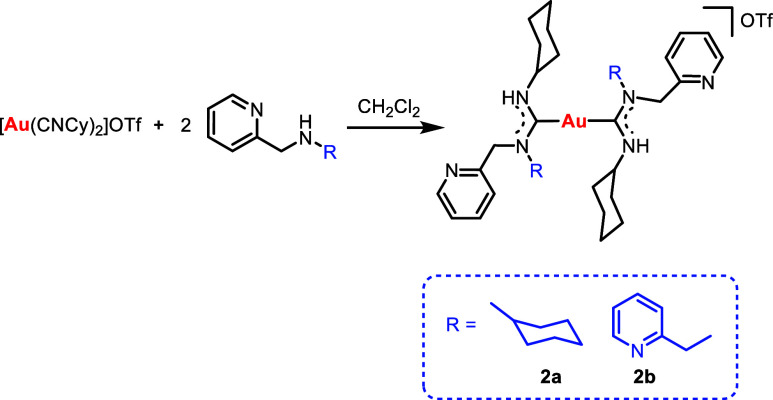
Synthesis of the Bis(carbene) Gold(I) Species **2**

Bis(carbene) gold complexes were achieved through the reaction of the complex [Au(CNCy)_2_]OTf with the corresponding amines (Scheme 2). In this case, only two representative examples with the non-chiral pycolyl derivatives were afforded in order to avoid the presence of rotamers in the molecule.

The characterization of complexes **2a** and **2b** was performed using techniques similar to those applied to family 1. The NMR spectra (Figures S10 and S13) exhibit the expected resonances. Interestingly, the ^13^C resonance for the carbene carbon appears at 205.2 and 206.1, respectively, showing a downfield shift compared to the chloride derivatives and even greater deshielding the related [Au(NHC)_2_]^+^ complexes.^35,36^ The mass spectra reveal, as the most abundant peaks, the cationic [M-OTf]^+^ at 795.4383 for **2a** and 814.3740 for **2b**.

**Scheme 3:**
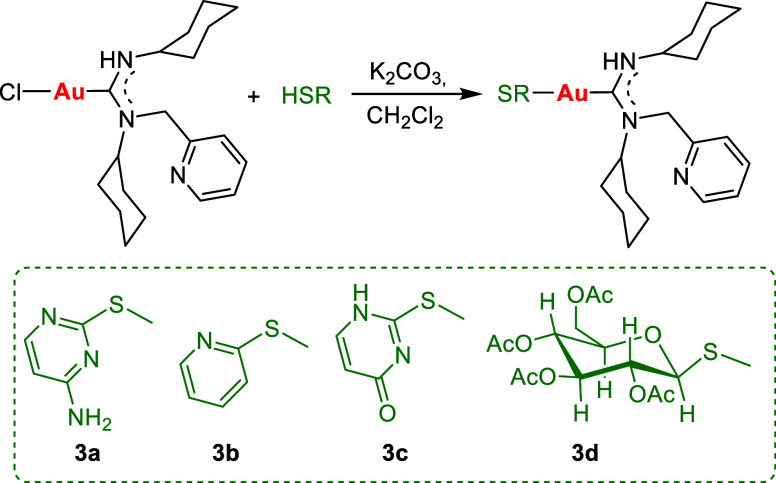
Synthesis of the Thiolate-Gold(I)-ADC Complexes **3**

The reactivity of the complex [AuCl{C(NHCy)(NCy-CH_2_py)}] (**1**) with various thiols was investigated to facilitate the formation of the corresponding gold(I) thiolates through a substitution reaction involving the chloride ligand. In the synthesis of gold(I) derivatives **3a–d**, a base, potassium carbonate, is used to help with the deprotonation of the 2-thiocytosine, 2-mercaptopyridine, 2-thiouracil, and 1-thio-β-d-glucose, thereby promoting the elimination of the HCl formed (Scheme 3).

Complexes **3a–d** were characterized by ^1^H and ^13^C{^1^H}-APT NMR spectroscopy (see Figures S14–S21), IR spectroscopy, and mass spectrometry. In the IR spectra, the disappearance of the ν(Au–Cl) band confirms the loss of the Au–Cl bond. The NMR spectra exhibit the expected resonances for the carbene ligand, along with additional signals corresponding to the incorporated thiolate ligands. The resonances for the carbene carbon atoms appear around 202 ppm, which is an intermediate chemical shift compared to those observed in Au–Cl or bis(carbene) gold derivatives and again downfield compared with the related [Au(SR)(NHC)] (182–185 ppm).^37^

### Crystal Structure Determination

**Figure 2:**
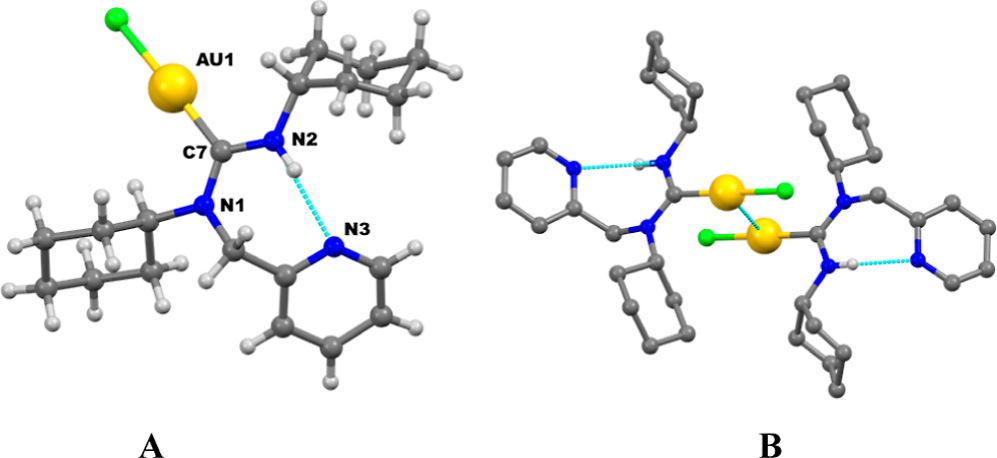
Molecular structure of complex **1a** (A) and association of molecules of **1a** in dimers through aurophilic interactions (B).

Suitable crystals for X-ray diffraction studies of complex **1a** were obtained through the slow diffusion of hexane into a dichloromethane solution of the complex. The compound crystallizes in the triclinic system within space group *P*1̅, featuring one molecule per asymmetric unit (Figure 2A). The gold atom exhibits a linear coordination typical of gold(I), although slightly distorted, as evidenced by the C7–Au1–Cl1 angle of 175.7(2)°. The Au–C and Au–Cl bond distances are 1.993(9) Å and 2.284(4), respectively, consistent with those found in other organometallic gold(I) chloride derivatives with carbene ligands.^34,35^ The bond lengths of the carbene group, N1–C7 at 1.333(12) Å and N2–C7 at 1.331(12) Å, clearly indicate their multiple bond character, while the N2–C7–N1 angle of 117.6(9)° approaches 120°, reflecting the sp^2^ hybridization of the carbene carbon.

Furthermore, as illustrated in Figure 2B, there is an intramolecular hydrogen bond between N3 of the pyridine and N2–H2A of the carbene group (N2–H2A···N3 at 2.088 Å, 147.05°). In addition, an intermolecular aurophilic interaction between the two gold atoms of two neighboring molecules can be observed, at a distance of Au1···Au1 (−*x*, −*y* + 1, −*z*) of 3.4384(9) Å, shorter than the sum of the van der Waals radii of the gold atoms.

A different structural form crystallized as a dichloromethane solvate from a different sample, also obtained by diffusion of hexane into a dichloromethane solution. In this case, the compound crystallized in the monoclinic system space group *I*2/*c*. Each asymmetric unit is composed of one molecule of compound **1a** and one molecule of dichloromethane. The molecule (Figure S22) is essentially the same as that in the triclinic crystal, with similar bond distances. An important difference is that in this crystal, there are no “aurophilic” interactions between the gold atoms; the closest ones are more than 5 Å apart. Perhaps the absence of these interactions causes the gold atom to be in a practically linear environment, less distorted than in the triclinic crystal, C7–Au1–Cl1 179.3(4)°. The existence of an intramolecular hydrogen bond of 2.131 Å between the pyridinic nitrogen and the NH of the carbene group is also observed.

**Figure 3:**
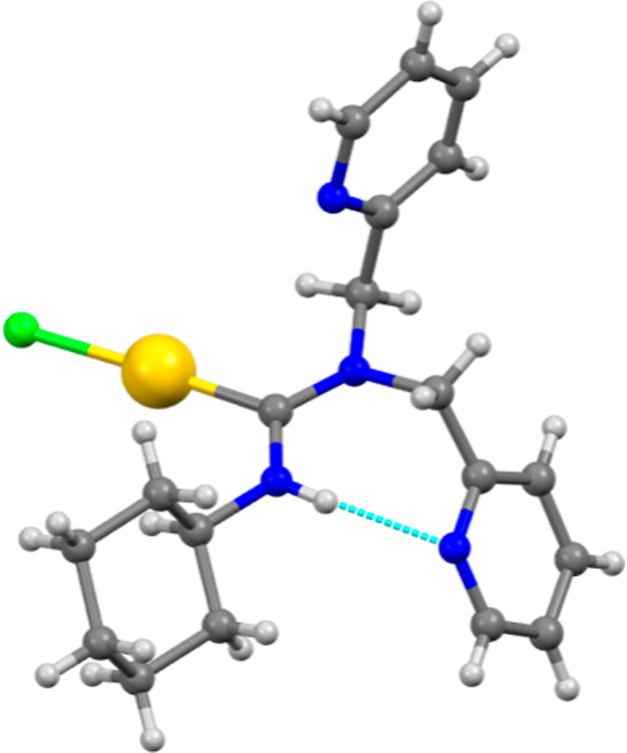
Molecular structure of complex **1b**.

The crystal structure of complex **1b** is shown in Figure 3. Its structural features closely resemble those of complex **1a**, with a nearly linear Cl1–Au–C1 angle of 177.23(9)° and a slightly longer Au–C1 bond length of 2.071(3) Å, likely attributed to the presence of more aromatic substituents on the nitrogen atom of the carbene moiety. Additionally, a short intramolecular N–H···N hydrogen bond of 2.143 Å is observed. Notably, there are no significant Au···Au interactions, with the shortest Au···Au distance measuring 5.381 Å.

**Figure 4:**
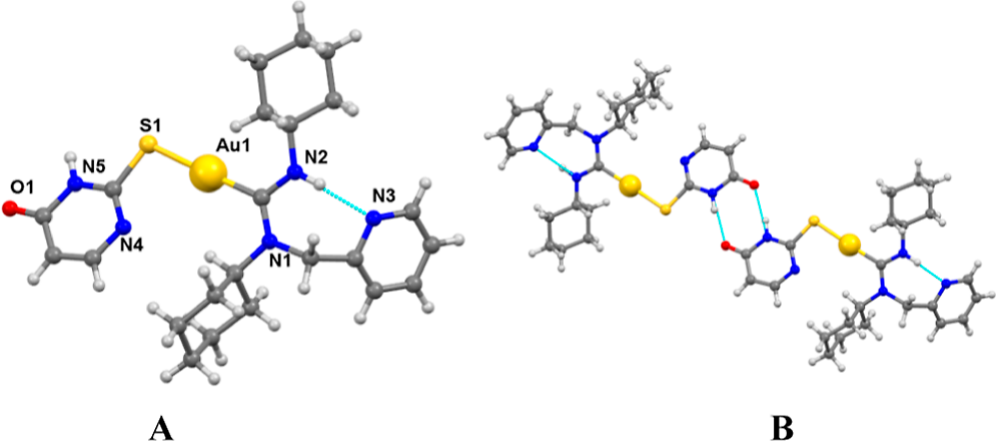
Molecular structure of complex **3c** (A) and association of molecules of **3c** in pairs through hydrogen bonding (B).

In the crystal structure of complex **3c** (Figure 4A), the gold atom adopts a slightly distorted linear geometry with a C1–Au1–S1 angle of 175.9(3)°. The Au–S (2.302(3) Å) and Au–C (1.962(15) Å) bond lengths are consistent with those observed in other derivatives containing the S–Au(I)–C fragment and are comparable to those in the parent complex **1a**. The C–N bond lengths in the carbene group (N1–C1:1.360(15) Å, N2–C1:1.366(15) Å) are shorter than typical single bonds, consistent with significant double-bond character.

The angles around the C1 atom of the carbene (112.5°, 123.4°, and 124.0°) indicate a somewhat distorted trigonal planar geometry, which is likely due to steric effects. No metal–metal interactions are present, but the molecules associate in pairs through intermolecular hydrogen bonding. In Figure 4B, hydrogen bonds between the nitrogen and oxygen atoms of the thiouracil group in one molecule and the oxygen and nitrogen atoms of the same group in another molecule are illustrated (N5–H5···O1 [−*x*, −*y*, −z]: 1.923 Å, 166.23°). Additionally, an intramolecular hydrogen bond is observed involving the NH group of the carbene and the nitrogen atom of the pyridine ring (N2–H1···N3: 2.003 Å, 147.56°).

### Cytotoxicity and Selectivity Studies

As an initial step in studying the cytotoxic properties of the complexes, stability studies were conducted to assess their behavior in biological media. First, the stability was studied by NMR (Figures S23–S32), revealing that the complexes remain stable in DMSO solution over extended periods. This stability is attributed to the low affinity of gold for oxygen-donor ligands, preventing exchange reactions with the solvent. Second, solutions of the target compounds were prepared in DMSO and then diluted in phosphate-buffered saline (PBS). UV–visible measurements were taken at multiple time points over a 24 h period, while the solutions were incubated at 37 °C to monitor for any changes or formation of new species in the assay medium. All compounds tested demonstrated stability under these simulated biological conditions (Figures S33–S42). The most distinctive feature is the absence of the plasmon band (500–600 nm) associated with gold nanoparticles, confirming the stability of the complexes in solution.

To evaluate the potential anticancer properties of the gold complexes, four cancer cell lines representing some of the most aggressive cancers were selected: A549 lung carcinoma, MiaPaca2 pancreatic cancer, HCT116 WT colon adenocarcinoma, and Jurkat T-cell leukemia (Table 1).

**Table 1:** IC_5O_ Values for Complexes **1–3** in the Different Cancer Cells Measured at 24 h (μM)

	A549	HCT116 WT	Jurkat	MiaPaca2
CisPt^b^	114 ± 9	1.2 ± 0.1	10.8 ± 1.2	76.5 ± 7.4
AF^a^^,^^b^	7.59	7.8 ± 0.9	1.4	2.34
**1a**	4.3 ± 0.7	2.45 ± 0.07	0.8 ± 0.1	3.1 ± 0.8
**1b**	9.1 ± 1.6		4.1 ± 0.9	8.6 ± 1.9
**1c**	1.3 ± 0.3	9.6 ± 1.7	6.76 ± 0.33	3.4 ± 0.5
**1d**	21.6 ± 1.4	2.1 ± 0.3	8.1 ± 1.3	9.9 ± 1.1
**2a**	13.4 ± 1.0	0.61 ± 0.11	0.65 ± 0.01	1.75 ± 0.15
**2b**	0.82 ± 0.05	1.1 ± 0.3	0.50 ± 0.02	0.38 ± 0.10
**3a**	0.43 ± 0.01	0.95 ± 0.14	5.2 ± 0.9	3.30 ± 0.25
**3b**	2.3 ± 0.5	4.22 ± 0.25	0.79 ± 0.03	2.0 ± 0.5
**3c**	2.04 ± 0.19	2.7 ± 0.3	3.7 ± 0.4	1.60 ± 0.17
**3d**	1.30 ± 0.12	1.09 ± 0.03	0.26 ± 0.03	1.5 ± 0.2

a AF auranofin.

b Literature values.^38−43^

The data revealed substantial cytotoxicity across all measured compounds, highlighting distinct activity profiles within each family. In the first family, where chloride serves as the auxiliary ligand, compound **1a** displayed excellent activity across all cell lines, being more active against leukemia cells. In contrast, compound **1b** exhibited lower activity overall but was also more active against leukemia cells. Among the chiral compounds, aminoindanol-substituted variant **1c** exhibited selectivity for lung and pancreatic cancer, while compound **1d** targeted more selectively colon cancer. This diversity highlighted the critical role played by the ADC ligand.

The bis(carbene) derivatives showed remarkable cytotoxic activity, likely due to their cationic nature, which could enhance cellular uptake. Gold(I) thiolate-carbenes emerged as especially potent, with compounds bearing thiocytosine (**3a**) and thioglucopyranosato (**3d**) ligands demonstrating IC_50_ values in the submicromolar range.

Overall, gold(I) thiolate and gold bis(carbene) complexes exhibited superior cytotoxicity compared with other Au(I) complexes with chloride ligands. This emphasized the critical impact of ligand choice on cytotoxic efficacy.

We extended our study to healthy human lymphocytes donated by the Blood and Tissue Bank of Aragon, to assess the selectivity of our compounds by comparing IC_50_ values between lymphocytes and Jurkat cells. This approach allowed us to determine the tumor selectivity of each compound. Notably, all compounds except complex **3c** displayed greater toxicity toward tumor cells than toward healthy cells. Complexes **1d**, **2a**, **3d**, and especially **3b** stood out, demonstrating exceptionally high selectivity and remarkably low IC_50_ values in tumor cells. Table 2 provides a detailed comparison of IC_50_ values for Jurkat cells and healthy lymphocytes along with the selectivity index for each compound.

**Table 2:** IC_5O_ Values for Complexes **1–3** in the Jurkat Cancer Cells and Healthy Lymphocytes Measured at 24 h (μM) and Selectivity Index (SI)

	Jurkat	lymphocytes	SI
**1a**	0.82 ± 0.13	3.86 ± 0.25	4.70
**1c**	6.76 ± 0.33	10.64 ± 1.12	1.57
**1d**	8.1 ± 1.3	49.4 ± 1.2	**6.13**
**2a**	0.65 ± 0.01	5.9 ± 0.7	**9.01**
**2b**	0.50 ± 0.02	1.51 ± 0.16	3.02
**3a**	5.2 ± 1.0	11.7 ± 1.2	2.27
**3b**	0.79 ± 0.03	58.2 ± 0.9	**73.68**
**3c**	3.7 ± 0.4	0.52 ± 0.11	0.14
**3d**	0.26 ± 0.03	2.19 ± 0.01	**8.42**

### Studies of the Mechanism of Cell Death

Apoptosis, a “clean” form of cell death that does not trigger an inflammatory response, is particularly desirable in anticancer treatments as apoptotic cells undergo orderly destruction while preserving cell membrane integrity. To investigate whether our compounds induce apoptosis or other types of cell death, we conducted MTT assays on two cell lines: HCT116 WT and HCT116 double knockout (DKO), with the latter being a human colorectal carcinoma strain resistant to mitochondrial apoptosis. By comparing the IC_50_ values in these two lines, we aimed to determine the mechanism of action of our compounds (Table 3).

**Table 3:** IC_5O_ Values for Complexes **1–3** in the HCT116 WT an DKO Cancer Cells Measured at 24 h (μM)

	HCT WT	HCT DKO
**1a**	2.45 ± 0.07	3.7 ± 1.0
**1c**	9.6 ± 1.7	13.4 ± 1.2
**1d**	2.1 ± 0.3	2.9 ± 0.6
**2a**	0.61 ± 0.11	1.5 ± 0.2
**2b**	1.1 ± 0.3	0.58 ± 0.06
**3a**	0.95 ± 0.14	3.0 ± 0.6
**3b**	4.22 ± 0.25	4.5 ± 0.8
**3c**	2.7 ± 0.30	5.9 ± 0.9
**3d**	1.09 ± 0.03	1.59 ± 0.13

The results presented below show relatively similar IC_50_ values for HCT116 WT and DKO cells, suggesting that our compounds act through pathways other than apoptosis. IC_50_ values for the DKO line are generally slightly higher than those for the WT line, although the difference is minor. This suggests that while our compounds may activate apoptosis, they also likely trigger alternative cell death mechanisms independent of the Bax and Bak proteins, given the typically high resistance of HCT116 DKO cells to apoptotic pathways.

To gain deeper insights into the type of cell death induced by the gold(I) complexes, we conducted a flow cytometry analysis. This approach allowed us to specifically detect apoptosis by examining the rearrangement of membrane phospholipids that occurs at the onset of apoptosis. During this process, phosphatidylserine (PS), normally located on the inner membrane surface, translocates to the outer membrane, exposing it to the external environment. To identify apoptotic cells, Annexin V, a protein with a high affinity for PS, which binds selectively to cells displaying PS on their outer membrane in a calcium-dependent manner, was used. Annexin V was conjugated with a fluorochrome (DY-634), allowing us to accurately quantify the degree of apoptosis within the cell population through fluorescence detection.

**Figure 5:**
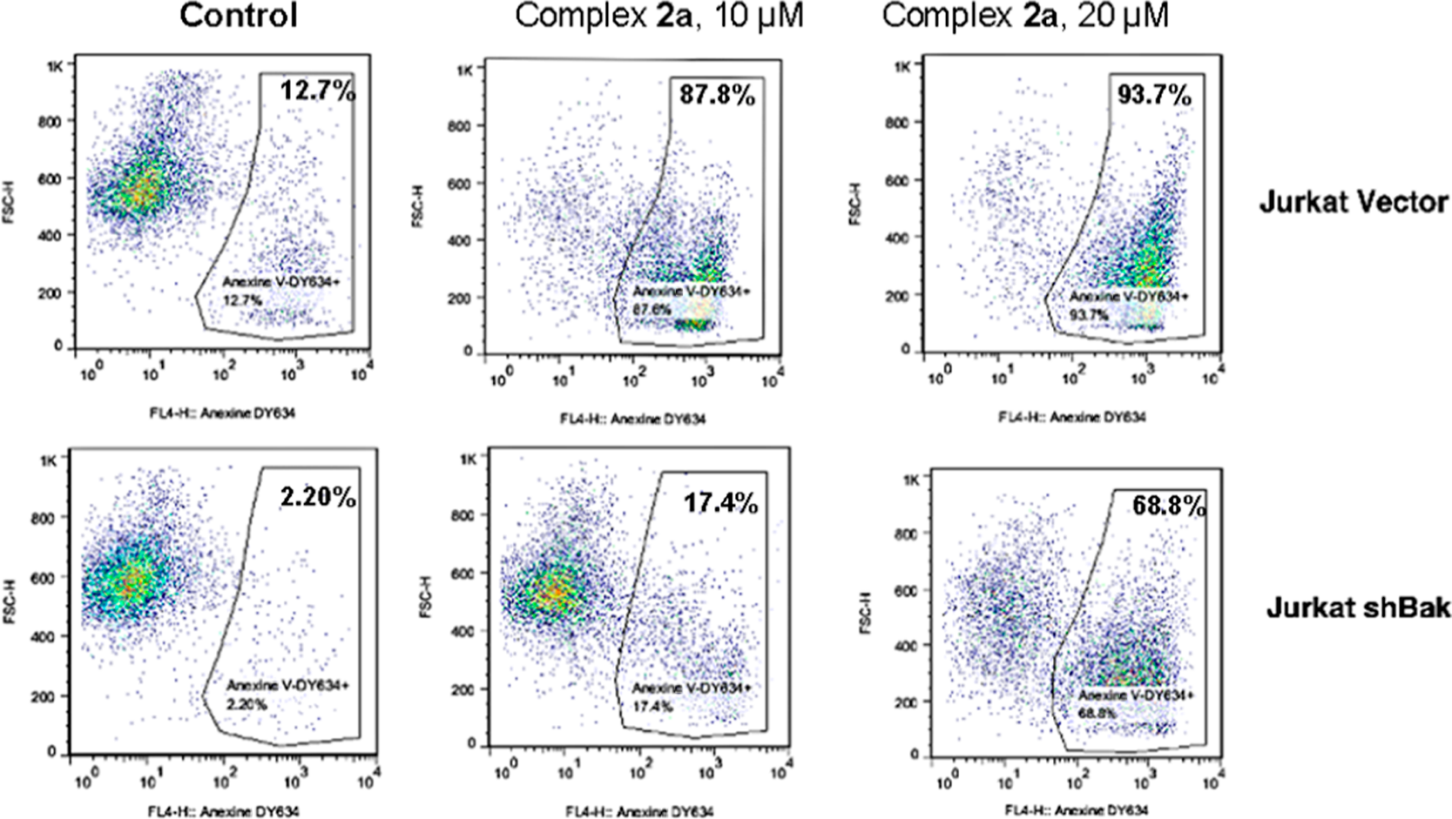
Flow cytometry studies on complex **2a** in Jurkat Vector and Jurkat shBak at 10 μM and 20 μM.

The study was conducted with two different concentrations of 10 and 20 μM (high concentrations were used to ensure significant cell death rather than merely inhibiting cell growth) on the Jurkat cell line and on Jurkat shBak, a strain resistant to mitochondrial apoptosis (Figure 5).

Observing the graphs, we could conclude that at low doses, the Jurkat shBak cell line is somehow protected compared to the Jurkat vector line, which means that cell death is probably due to mitochondrial apoptosis. However, at high doses, there are hardly any differences, which would mean that other mechanisms of cell death are activated at high concentrations.

**Figure 6:**
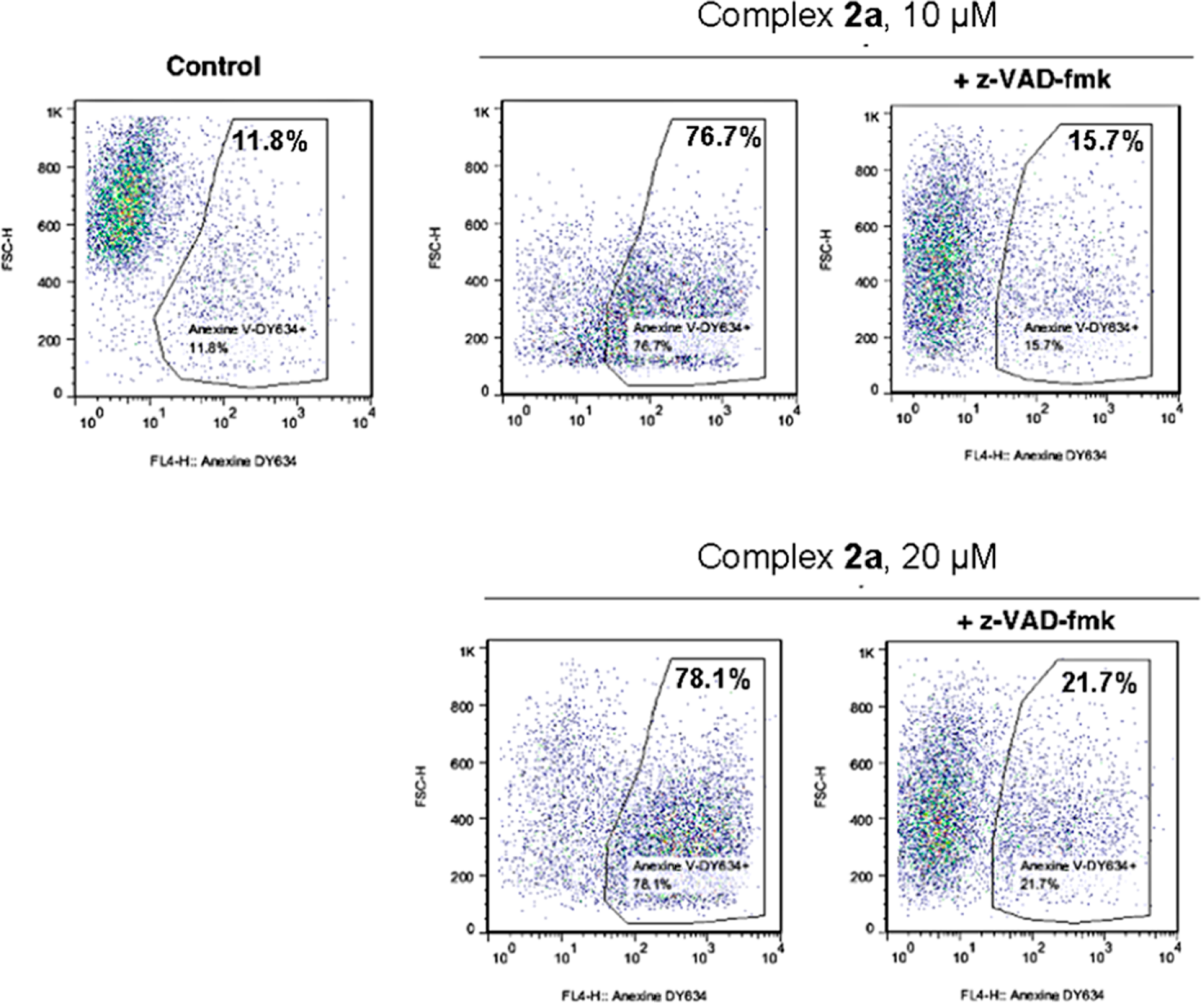
Flow cytometry studies on complex **2a** in Jurkat and Jurkat + z-VAD-fmk at 10 μM and 20 μM.

To confirm further these findings, we conducted flow cytometry experiments with and without caspase inhibitor z-VAD-fmk (Figure 6). Caspases are crucial enzymes in the apoptosis pathway, so a reduction in apoptosis in the presence of this inhibitor would indicate that cell death is mediated by caspase-dependent apoptosis. The results were striking as in the control group cells maintained a uniform size, but upon treatment with compound **2a**, cell size dramatically decreased, signaling cell death. At lower concentrations, the caspase inhibitor z-VAD-fmk significantly protected cells from apoptosis, as indicated by most cells falling outside the positive zone. However, at higher concentrations, this protective effect diminished.

**Figure 7:**
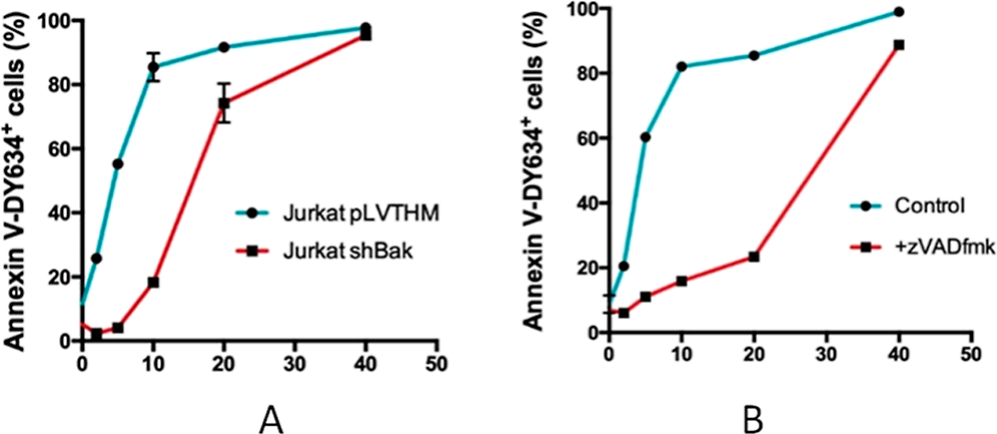
Graphics for the flow cytometry studies on complex **2a** at different concentrations: (a) Jurkat Vector and Jurkat shBak and (b) Jurkat and Jurkat + z-VAD-fmk.

The graphics presented in Figure 7 illustrate the conclusions drawn from these two studies. First, in Jurkat and Jurkat shBak cells, it is evident that at high doses, there are minimal differences between the two cell lines. This suggests that other mechanisms of cell death are activated at higher concentrations. Jurkat shBak shows protective effects at concentrations lower than 20 μM, but this protective effect diminishes at higher concentrations, as reflected in the decreasing gap between the two graphs (Figure 7A). Second, Figure 7B further highlights this trend, demonstrating a pronounced difference between the control and z-VAD-fmk curves at low concentrations. However, this difference narrows at higher concentrations. These findings indicate that at low concentrations, cell death is predominantly driven by caspase-dependent mitochondrial apoptosis, whereas at higher concentrations, alternative cell death pathways become active.

A similar study was performed for the thiolate complex **3d** obtaining similar results. In this case, the difference between the cell lines Jurkat and Jurkat shBak was not significant, and the same observation occurred in the presence of z-VAD-fmk, indicating that cell death was caused by apoptosis but is independent of caspase. The data can be found in the Supporting Information (Figures S43–S45).

### Flow Cytometry Studies

#### Cell Cycle Arrest Studies and Cell Death

The cell cycle is divided into two primary phases: mitosis (M), where cell division occurs, and interphase, which encompasses the G1 (pre-DNA synthesis), S (DNA synthesis), and G2 (predivision) stages. After interphase, cells may enter the G0 phase, a state where they are not actively cycling but maintain the potential for division. The G0 phase mostly includes nongrowing or nonproliferating cells, which can revert to the G1 phase in response to proliferation signals or other mitogenic stimuli. The progression of the cell cycle is regulated by CDK-mediated phosphorylation, protein degradation via the ubiquitin–proteasome system, and checkpoints at crucial transitions (G1-S and G2-M) to ensure genomic integrity. The p53 gene plays a pivotal role in halting the cycle by inhibiting cyclins and is closely linked to apoptosis following cycle arrest. Investigating the cell cycle is vital for understanding drug mechanisms. Techniques such as flow cytometry, which measures DNA content, are used to analyze the distribution of cells across different cycle phases. Thus, A549 cells were treated with compound **3b** for 24 h to study its effects on both in the cell cycle and in the type of cell death (Table 4).

**Table 4:** Cell Cycle and Type of Cell Death Induced by Compound **3b** after 24 h of Incubation

cell cycle	G0G1	S	G2M
control	58.82	28.73	12.44
**3b**	82.85	8.60	8.55

cell death	intact (A3)	L-AP^a^(A2)	E-AP^a^ (A4)	N^a^ (A1)
control	70.57	27.88	0.58	0.97
**3b**	25.73	59.65	11.20	3.42

a E-AP: early apoptosis, L-AP: late apoptosis, N: necrosis.

As shown in Table 4 and Figures S46–S48, the presence of compound **3b** significantly increased the number of cells in the G0/G1 phase and decreased S and G2/M phases compared to the control. These data suggest that G0/G1 phase arrest may be responsible for the antiproliferative effects of compounds **3b** on A549 cells. Arrest in the G0/G1 phase can result from severe and irreparable DNA damage, preventing the cell from entering the S phase. In addition, **3b** preferentially promotes cell death by triggering apoptosis.

#### ROS Production and Mitochondrial Membrane Potential

**Figure 8:**
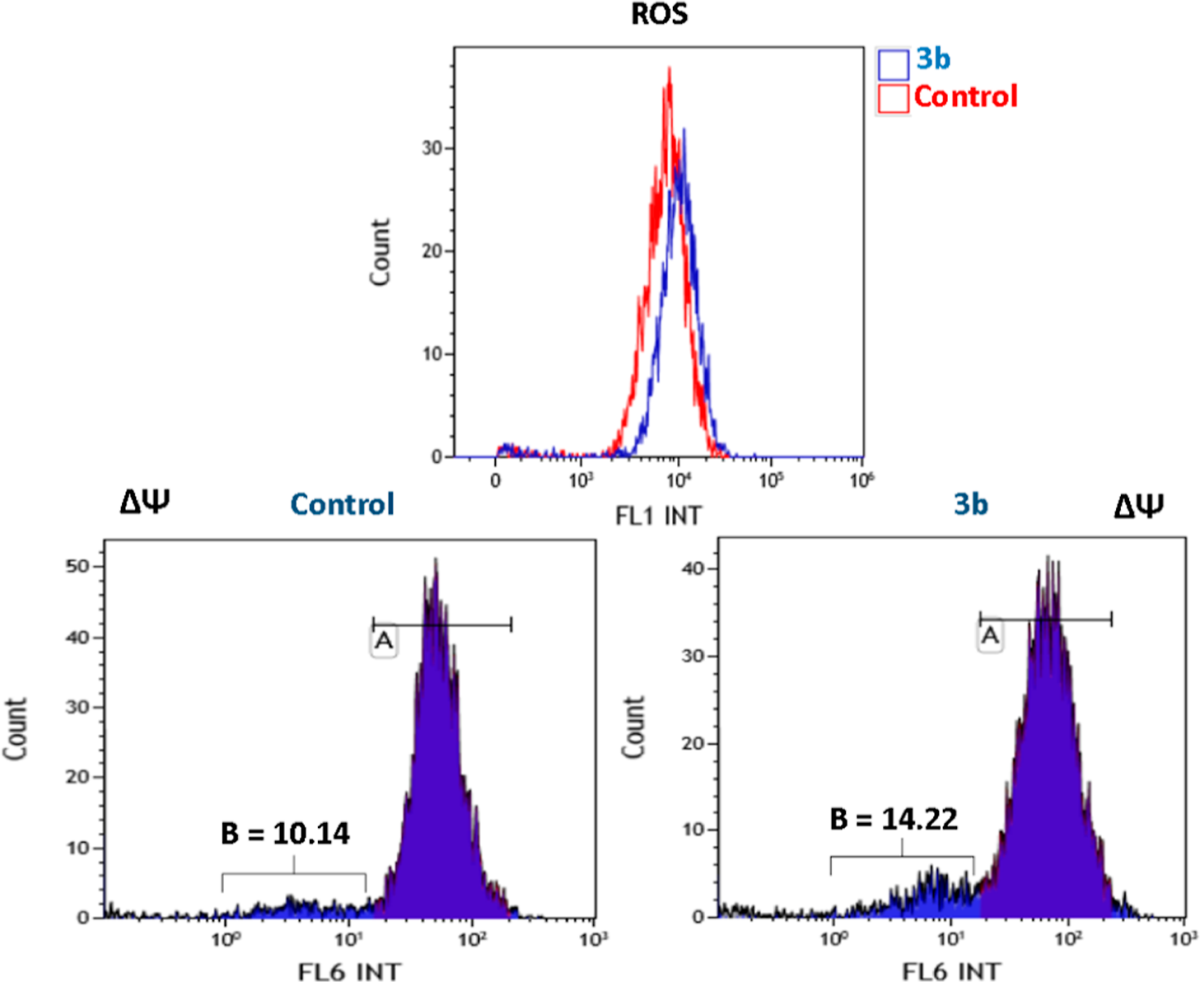
ROS and ΔΨ of complex **3b** in A549 cells.

Redox homeostasis is crucial for sustaining cellular processes, and its disruption can lead to an increase in the level of reactive oxygen species (ROS). Elevated ROS levels cause oxidative stress, which can damage proteins and DNA, ultimately triggering cell death. This process is often associated with programmed cell death mechanisms, such as apoptosis or autophagy. Mitochondria play a central role in regulating both cell survival and death. As critical hubs of metabolic activity, they are implicated in various diseases and have become a major therapeutic target, especially in cancer treatment. Mitochondrial membrane potential (ΔΨ), maintained by ion concentration gradients, is a key indicator of mitochondrial function. Disruption in energy metabolism reduces ΔΨ, signaling mitochondrial damage. Therefore, the ability of compound **3b** to generate ROS and mitochondrial membrane depolarization was evaluated. Figure 8 (top) illustrates a slight increase in the reactive oxygen species, as indicated by the enhanced luminescence intensity. This shift appears to the right, with the control shown in red and **3b** in blue. Additionally, a mild decrease in the mitochondrial membrane potential is observed (Figure 8, bottom). In the control group, 10.14% of the mitochondrial membrane potential is disrupted, whereas, in the presence of complex **3b**, this disruption increases to 14.22%. These effects were observed after incubating A549 cells with **3b** for 24 h.

### DNA Interaction

The interaction of the bis(carbenes) (**2a** and **2b**) with DNA was studied due to their potentially more planar structure, which has been associated with DNA intercalation. These compounds might enable DNA intercalation. Absorption spectral titration experiments were performed according to the methodology described in our previous works.^44,45^

**Figure 9:**
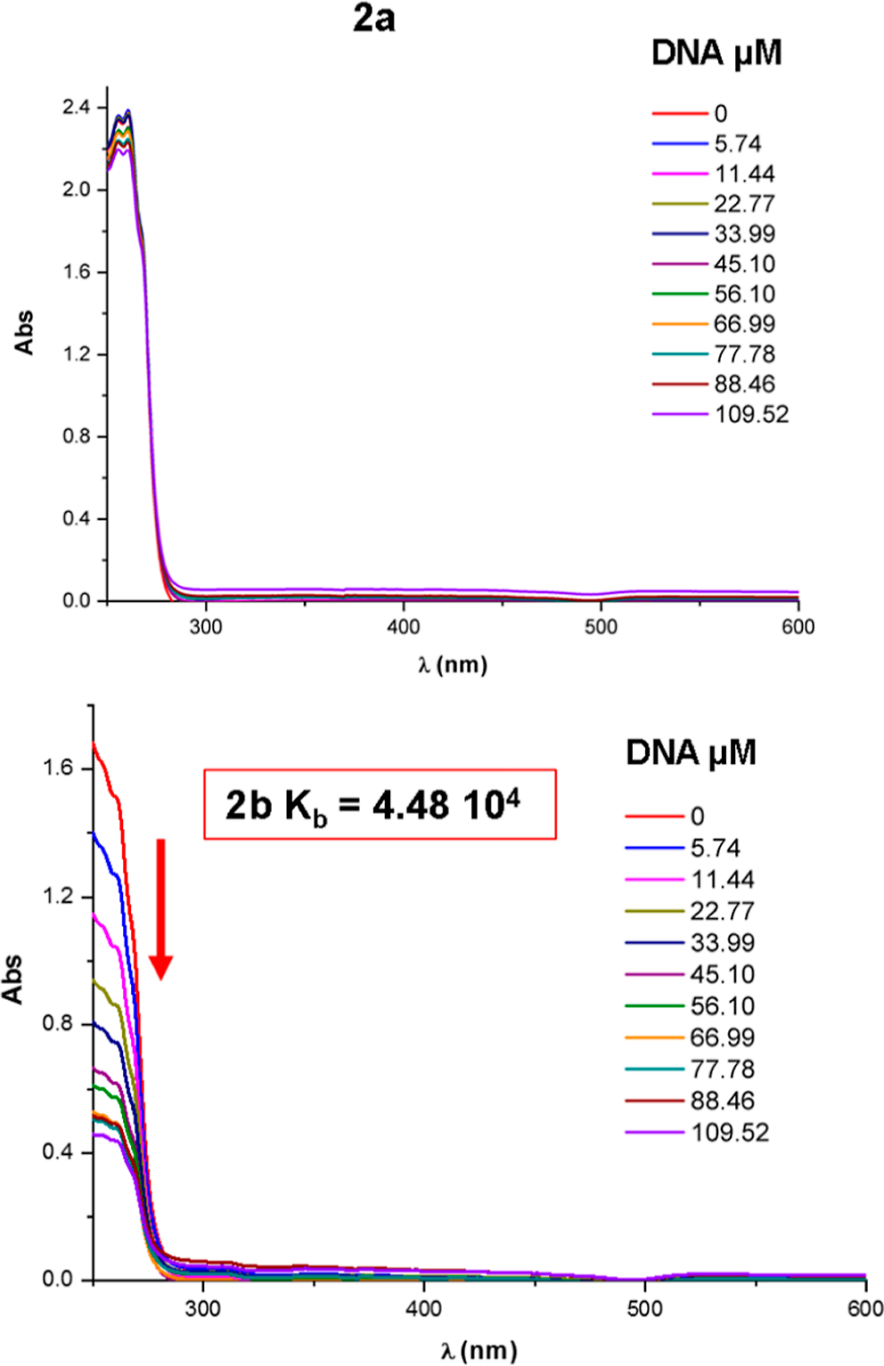
Absorption spectral titration experiments for complexes **2a** and **2b**. The arrow indicates that the absorbance of the complex decreases with the addition of CT-DNA.

Figure 9 presents the UV–vis spectra obtained by gradually adding increasing amounts of DNA to a solution of **2a** or **2b** (100 μM), along with data extracted from the absorption spectral titration experiments. As shown in Figure 9, compound **2a** exhibits minimal interaction with DNA, resulting in negligible changes in the absorption spectra. Consequently, this minimal interaction precluded calculation of the DNA-binding constant (*K*_b_). In contrast, compound **2b** exhibits a clear decrease in band intensity with increasing DNA concentration, indicating that it interacts with DNA via intercalation. The calculated binding constant (*K*_b_ = 4.48 × 10^4^) suggests that **2b** is a moderate intercalator. These results align with the structural characteristics of these compounds. For compound **2b**, the presence of pyridine rings enhances its planarity, unlike compound **2a**, where the higher number of cyclohexyl rings reduces planarity.

### Inhibition Studies of TrxR in A549 Cell Lysates

**Figure 10:**
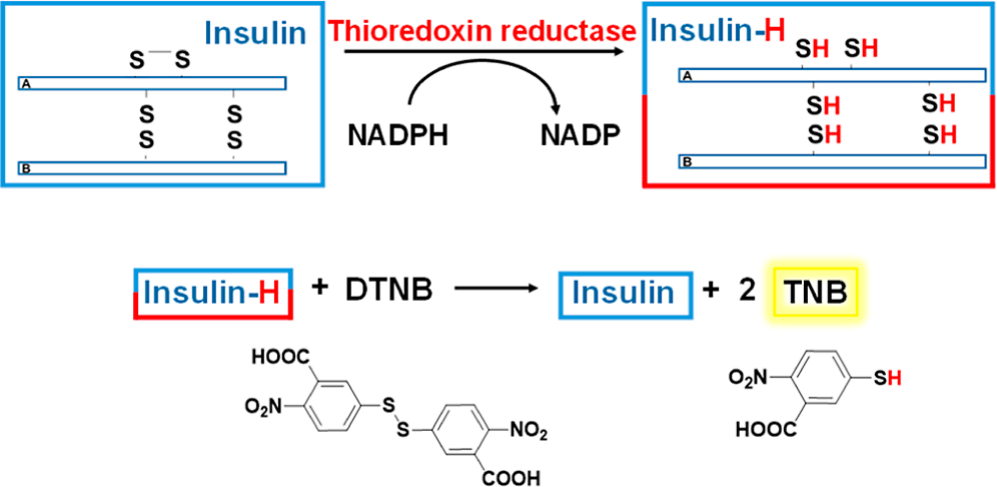
Insulin reduction assay.

Thioredoxin Reductase (TrxR) is a critical biological target of gold(I) complexes. Therefore, the ability of the selected compounds (**2b**, **3b**, and **3d**), along with auranofin as a reference, to inhibit the TrxR system was evaluated using an insulin reduction assay. This assay is well established for assessing enzyme inhibition.^46^ The thioredoxin reductase system is known to efficiently reduce insulin interchain disulfide bridges, making insulin an ideal substrate for this analysis. Additionally, the reaction can be monitored using DTNB, which reacts with free thiol groups of insulin to generate TNB, which is a yellow compound. This allows the inhibition of TrxR to be quantified by UV–vis spectroscopy (Figure 10).

The inhibition assay was conducted using the A549 cell line, which overexpresses TrxR.^47^ Both the selected compounds and auranofin were tested at a concentration of 3 μM. The results are presented in Table 5.

**Table 5:** Inhibition Studies of Thioredoxin Reductase Activity (%)

compound	inhibition of TrxR %
auranofin	85 ± 3
**2b**	73 ± 2
**3b**	51 ± 5
**3d**	44 ± 5

The results of this study indicate that complex **2b**, a bis(carbene) compound, exhibits inhibition levels comparable to those of auranofin at the same concentration. In contrast, complexes **3b** and **3d**, which feature thiolate as the auxiliary ligand, demonstrate lower inhibition than auranofin, with approximately half of the inhibitory effect at this concentration. It is important to highlight that the results obtained in this study are highly significant, as all the evaluated compounds have demonstrated potent inhibition of the TrxR system in cell lysates. Notably, compound **2b** stands out, with an inhibition comparable to that of auranofin, which is one of the most potent and selective TrxR inhibitors known to date. Furthermore, it is important to highlight that, to date, Au-ADC compounds have demonstrated poor inhibition of the TrxR system.^20^

## Conclusions

In this study, the successful synthesis and evaluation of the anticancer properties of a novel series of gold(I) ADC complexes have been accomplished, significantly advancing the underexplored potential of these compounds in biological systems. Although such gold complexes are well established in catalysis and materials science, this work highlights their transformative potential in anticancer therapeutics. Through the nucleophilic addition of various amines to isocyanide [AuCl(CNCy)] complexes, three distinct families of compounds were developed based on their auxiliary ligands: gold-chloride ADC complexes (featuring chiral and achiral substituents), bis(carbene) complexes, and thiolate-gold ADC complexes. Comprehensive characterization via NMR, IR, HRMS, and X-ray crystallography (for compounds **1a**, **1b**, and **3c**) validated their structures.

The findings indicate that ligand design critically determines the cytotoxic efficacy of gold(I) ADC complexes. Among the tested compounds, gold-chloride derivatives exhibited selective cytotoxicity, with **1a** and **1b** showing leukemia-specific potency and **1a** being more active in general. Chiral derivatives like **1c** exhibited targeted effects against lung and pancreatic cancers and **1d** toward colon cancer. Bis(carbene) complexes (**2a** and **2b**) displayed remarkable cytotoxicity, likely enhanced by their cationic nature, which facilitates cellular uptake. Remarkably, the gold(I) thiolate-carbenes emerged as the most potent subgroup, achieving submicromolar IC_50_ values, with compound **3b** attaining a selectivity index (SI) of 73.68 compared to healthy cells.

Mechanistic studies revealed that these complexes preferentially induce apoptosis at low concentrations, while high concentrations trigger alternative cell death pathways. Cell cycle analysis showed that **3b** arrested cells in the G0/G1 phase, indicating severe DNA damage. Interestingly, bis(carbene) complexes **2** exhibit DNA-binding properties, with **2b** bearing two pyridyl substituents showing the highest constant. Additionally, compound **3b** moderately increased the level of ROS generation and mitochondrial membrane depolarization. TrxR inhibition studies identified **2b**, **3b**, and **3d** as potent inhibitors, with **2b** matching the potency of auranofin, which is one of the best TrxR inhibitors.

In conclusion, the synthesized gold ADC complexes represent a significant advancement in the development of chemotherapeutic agents, combining high selectivity with potent cytotoxic activity against resistant cancer cell lines. Their multitarget mechanisms, including ROS generation, mitochondrial damage, DNA binding, and TrxR inhibition, highlight their versatility in disrupting cancer cell survival. These findings not only underscore the therapeutic potential of gold ADC complexes but also pave the way for further exploration of their structure–activity relationships and optimization as effective anticancer agents.

## Experimental Section

### Synthesis and Characterization

#### General Procedure for the Synthesis of **1a–1d**

To a solution of [AuCl(CNCy)] in dichloromethane (25 mL) was added 1 equiv of the corresponding amine: (*N*-(pyridylmethyl)cyclohexane) (**1a**), di(2-picolyl)amine (**1b**), (1*R*,2*S*)-(+)-*cis*-1-amino-2-indanol (**1c**), (1*R*,2*R*)-(−)-*N*-*p*-tosyl-1,2-cyclohexanediamine (**1d**), and the mixture was stirred at room temperature during 24 h, The solvent was removed under vacuo to 5 mL and *n*-hexane was used to precipitate the solid. After filtration, compounds **1a–1d** were obtained.

**1a**: [AuCl(CNCy)] 0.1024 g (0.3 mmol) and *N*-(pyridylmethyl)cyclohexane 0.0570 g (0.3 mmol). Yield = 78%. ^1^H NMR (CD_2_Cl_2_, 400 MHz) δ: 8.79 (s, NH), 8.51 (d, CH_Ar,2_), 7.76 (t, CH_Ar,4_), 7.36 (d, CH_Ar,5_), 7.28 (t, CH_Ar,3_), 4.86–4.74 (m, H_cy_), 4.14–4.05 (m, CH_cy_), 2.03–1.02 (m, CH_2_,_cy_). ^13^C{^1^H}-APT NMR (CD_2_Cl_2_, 101 MHz) δ: 192.8 (s, C_carbene_), 156.5 (s, C_ipso,py_), 149.0 (s, CH_py_), 138.4 (s, CH_py_), 123.8 (s, CH_py_), 123.7 (s, CH_py_), 69.6 (s, CH_cy_), 59.9 (s, CH_cy_), 51.0 (s, CH_2_), 34.4 (s, CH_2,cy_), 32.8 (s, CH_2,cy_), 26.3 (s, CH_2,cy_), 25.9 (s, CH_2,cy_), 24.9 (s, CH_2,cy_). Elemental analysis (%): C_19_H_29_AuClN_3_ requires: C 42.91, H 5.50, N 7.90; found: C 43.14, H 5.35, N 7.69. HRMS (ESI-QTOF) *m*/*z* (%): Calculated for C_19_H_29_AuClN_3_Na, 554.1608; found, 554.1604 [M + Na]^+^. IR: ν (C=N): 1587 cm^–1^, ν (NH): 2932 cm^–1^, ν (Au–Cl): 325 cm^–1^.

**1b**: [AuCl(CNCy)] 0.1024 g (0.3 mmol) and di(2-picolyl)amine 0.0598 g (0.3 mmol). Yield = 50%. ^1^H NMR (400 MHz, CDCl_3_) δ: 9.16 (m, 1H, NH), 8.57 (d, 1H, *J* = 4.2 Hz, H-6), 8.48 (d, 1H, *J* = 4.1 Hz, H-6′), 7.60 (m, 4H, H-3, H-3′, H-4, H-4′), 7.25 (m, 2H, H-5, H-5′), 7.00 (d, 1H, *J* = 7.7 Hz, H-3 o H-3′), 5.35 (s, 2H, CH_2_), 4.55 (s, 2H, CH_2_), 4.22 (m, 1H, CH_cy_), 2.02 (m, 2H, cy), 1.69 (m, 3H, cy), 1.43 (m, 4H, cy), 1.26 (m, 1H, cy). ^13^C{^1^H}-APT NMR (CDCl_3_, 101 MHz) δ: 193.0 (s, 1C, C=N), 156.5 and 154.8 (s, 2C, C-2, C-2′), 149.4 and 148.6 (s, 2C, C-6, C-6′), 137.9 and 137.2 (s, 2C, C-4, C-4′), 124.0 and 123.2 (s, 2C, C-3, C-3′), 123.6 and 123.7 (s, 2C, C-5, C-5′), 64.8 (s, 1C, CH_2_), 59.2 (s, 1C, CH_cy_), 55.3 (s, 1C, CH_2_), 34.0 (s, 2C, cy), 25.4 (s, 1C, cy), 24.4 (s, 2C, cy). Elemental analysis (%): C_19_H_24_AuClN_4_ requires: C 42.19, H 4.47, N 10.36; found: C 42.27, H 4.68, N 10.51. HRMS (ESI^+^) *m*/*z* %: Calculated for C_19_H_25_AuN_4_, 506.1739; found, 506.1800 [M – Cl]^+^.

**1c**: [AuCl(CNCy)] 0.0683 g (0.2 mmol) and (1*R*,2*S*)-(+)-*cis*-1-amino-2-indanol 0.0299 g (0.2 mmol). Yield = 59%. ^1^H NMR (CD_2_Cl_2_, 400 MHz) δ: 7.32–7.22 (m, CH_Ar_), 4.31 (m, CH_2,Ar_), 3.96–3.87 (m, CH_2cy_), 3.05 (m, CHNH), 2.82 (m, CHOH), 2.85 (s, OH), 2.22–1.90 (s, CH_2,cy_), 1.86–1.66 (s, CH_2,cy_), 1.53–1.36 (s, CH_2,cy_). ^13^C{^1^H}-APT NMR (CD_2_Cl_2_, 101 MHz) δ: 128.3 (s, CH_Ar_), 127.3 (s, CH_Ar_), 125.8 (s, CH_Ar_), 124.4 (s, CH_Ar_), 73.3 (s, CHOH), 59.3 (s, CHNH), 55.6 (s, CH_ipso_), 39.9 (s, CH_2cy_), 32.1 (s, CH_2,cy_), 25.1 (s, CH_2,cy_), 23.2 (s, CH_2,cy_). Elemental analysis (%): C_16_H_22_AuClN_2_O requires: C 39.16, H 4.52, N 5.71; found: C 38.89, H 4.34, N 5.89. HRMS (ESI-QTOF) *m*/*z* (%): Calculated for C_16_H_22_AuN_2_O, 455.1392; found, 455.1391 [M – Cl]^+^. IR: ν (C=N): 1567 cm^–1^, ν (NH): 3314, 2933 cm^–1^, ν (Au–Cl): 310 cm^–1^.

**1d**: [AuCl(CNCy)] 0.0683 g (0.2 mmol) and (1*R*,2*R*)-(−)-*N*-*p*-tosyl-1,2-cyclohexanediamine 0.0537 g (0.2 mmol). Yield = 68%. ^1^H NMR (CD_2_Cl_2_, 400 MHz) δ: 7.89–7.83 (m, rotamer A, Ph), 7.77–7.70 (m, rotamer B, rotamer C, Ph), 7.38–7.28 (m, rotamer A, rotamer B, rotamer C, Ph), 4.00–3.79 (m, CH_cy_), 3.47–3.34 (m, CH_cy_), 3.29–3.00 (m, CH_cy_), 2.43 (s, rotamer B or C, CH_3_), 2.42 (s, rotamer B or C, CH_3_), 2.41 (s, rotamer A, CH_3_), 2.30–1.02 (m, CH_2,cy_). ^13^C{^1^H}-APT NMR (CD_2_Cl_2_, 101 MHz) δ: 144.4 (s, Ph), 144.3 (s, Ph), 139.0 (s, Ph), 138.8 (s, Ph), 138.5 (s, Ph), 130.4 (s, Ph), 130.3 (s, Ph), 127.5 (s, Ph), 127.3 (s, Ph), 127.1 (s, Ph), 64.1 (s, CH), 63.8 (s, CH), 59.4 (s, CH), 57.8 (s, CH), 57.4 (s, CH), 57.3 (s, CH), 54.9 (s, CH), 52.0 (s, CH), 35.3 (m, CH_2,cy_), 34.6 (m, CH_2,cy_), 34.5 (m, CH_2,cy_), 33.1 (m, CH_2,cy_), 32.5 (m, CH_2,cy_), 32.1 (m, CH_2,cy_), 31.8 (m, CH_2,cy_), 25.8 (m, CH_2,cy_), 25.7 (m, CH_2,cy_), 25.6 (m, CH_2,cy_), 25.4 (m, CH_2,cy_), 25.3 (m, CH_2,cy_), 25.1 (m, CH_2,cy_), 25.0 (m, CH_2,cy_), 24.8 (m, CH_2,cy_), 24.7 (m, CH_2,cy_), 24.5 (m, CH_2,cy_), 21.8 (s, 1C, CH_3_). Elemental analysis (%): C_20_H_31_AuClN_3_O_2_S requires: C 39.38, H 5.12, N 6.89, S 5.26; found: C 39.51, H 5.31, N 6.97, S 5.08. HRMS (ESI-QTOF) *m*/*z* (%): Calculated for C_20_H_31_AuN_3_O_2_S, 574.1797; found, 574.1829 [M – Cl]^+^. IR: ν (NH): 3084, 2926, 2855 cm^–1^, ν (Au–Cl): 312 cm^–1^.

#### General Procedure for the Synthesis of **2a** and **2b**

To a solution of [(CNCy)Au(CNCy)]OTf 0.2070 g (0.5 mmol) in dichloromethane (25 mL) was added 2 equiv of the corresponding amine: di(2-picolyl)amine (0.2000 g, 1.0 mmol) (**2a**) and (*N*-(pyridylmethyl)cyclohexane) (0.1903 g, 1.0 mmol) (**2b**), and the mixture was stirred at room temperature during 7 h. The solvent was removed in vacuo to 5 mL and compounds **2a** and **2b** were obtained, after filtration, using *n*-hexane as a precipitating agent.

**2a**: Yield = 68%. ^1^H NMR (CD_2_Cl_2_, 400 MHz) δ: 9.51 (s, NH), 8.49 (d, CH_Ar,2_), 8.47 (d, CH_Ar,2′_), 7.64 (t, CH_Ar,4_), 7.59 (t, CH_Ar,4′_), 7.26 (d, CH_Ar,3_), 7.19 (d, CH_Ar,3′_), 7.17 (d, CH_Ar,3′_), 6.69 (t, CH_Ar,5_), 5.15 (s, CH_2_), 4.47 (s, CH_2_), 4.00–3.86 (m, H_cy_), 1.97–1.16 (m, CH_2,cy_). ^13^C{^1^H}-APT NMR (CD_2_Cl_2_, 101 MHz) δ: 206.1 (s, C_carbene_), 156.3 (s, C_ipso,py_), 154.9 (s, C_ipso,py_), 150.2 (s, CH_py_), 148.9 (s, CH_py_), 138.4 (s, CH_py_), 137.4 (s, CH_py_), 124.5 (s, CH_py_), 124.1 (s, CH_py_), 123.6 (s, CH_py_), 122.3 (s, CH_py_), 63.9 (s, CH_2_), 59.0 (s, CH_cy_), 56.3 (s, CH_2_), 34.6 (s, CH_2,cy_), 25.6 (s, CH_2,cy_), 24.9 (s, CH_2,cy_). Elemental analysis (%): C_39_H_58_AuF_3_N_6_O_3_S requires: C 49.57, H 6.19, N 8.89, S 3.39; found: C 49.74, H 6.36, N 8.71, S 3.57. HRMS (ESI-QTOF) *m*/*z* (%): Calculated for C_38_H_58_AuN_6_, 795.4383; found, 795.4414 [M-OTf]^+^. IR: ν (C=N): 1547 cm^–1^, ν (NH): 2987 cm^–1^, ν (OTf): 1258, 1224, 1136, and 1029 cm^–1^.

**2b**: Yield = 81%. ^1^H NMR (CD_2_Cl_2_, 400 MHz) δ: 9.51 (s, NH), 8.53 (d, CH_Ar,2_), 7.80 (t, CH_Ar,4_), 7.42 (d, CH_Ar,5_), 7.33 (t, CH_Ar,3_), 4.57–4.51 (m, CH_cy_), 4.45 (s, CH_2_), 4.04–3.09 (m, CH_cy_), 2.07–1.09 (m, CH_2,cy_). ^13^C-APT NMR (CD_2_Cl_2_, 101 MHz) δ: 205.2 (s, C_carbene_), 156.3 (s, C_ipso,py_), 149.0 (s, CH_py_), 138.7 (s, CH_py_), 123.4 (s, CH_py_), 124.0 (s, CH_py_), 69.1 (s, CH_cy_), 59.3 (s, CH_cy_), 51.4 (s, CH_2_), 34.8 (s, CH_2,cy_), 32.8 (s, CH_2,cy_), 26.4 (s, CH_2,cy_), 25.7 (s, CH_2,cy_), 25.1 (s, CH_2,cy_). Elemental analysis (%): C_39_H_49_AuF_3_N_8_O_3_S requires: C 48.59, H 5.12, N 11.62, S 3.33; found: C 48.79, H 5.31, N 11.91, S 3.23. HRMS (ESI-QTOF) *m*/*z* (%): Calculated for C_38_H_49_AuN_8_, 814.3740; found, 814.3766. [M-OTf]^+^. IR: ν (C=N): 1588 cm^–1^, ν (NH): 2927 cm^–1^, ν (OTf): 1266, 1219, and 1030 cm^–1^.

#### General Procedure for the Synthesis of **3a–3d**

To a solution of complex **1a** (0.0530 g) (0.1 mmol) in dichloromethane (25 mL) was added an excess of K_2_CO_3_ (0.1400 g 1.5 mmol) and 1 equiv of the corresponding thiolate: 2-thiocytosine (0.0128 g) (**3a**), 2-mercaptopyridine (0.0111 g) (**3b**), 2-thiouracil (0.0128) (**3c**), and 1-thio-β-d-glucose (0.0364) (**3d**), and the mixture was stirred for 3 h. Subsequently, the reaction was filtered over Celite, the solvent was removed under vacuo to 5 mL, and compounds **3a–3d** were obtained, after filtration, using *n*-hexane as a precipitating agent.

**3a**: Yield = 65%. ^1^H NMR (CD_2_Cl_2_, 400 MHz) δ: 8.52 (d, CH_py,2_), 8.41 (s, NH), 7.85 (t, CH_Ar,4′_), 7.76 (t, CH_Ar,4_), 7.37 (d, CH_Ar,5_), 7.26 (t, CH_Ar,3_), 6.00 (d, CH_Ar,3′_), 5.00 (m, H_cy_),4.68 (s, NH), 4.44 (s, CH_2_), 4.26 (m, H_cy_), 2.10–1.04 (m, CH_2_,_cy_). ^13^C{^1^H}-APT NMR (CD_2_Cl_2_, 101 MHz) δ: 202.3 (s, C_carbene_), 162.5 (s, C_ipso,py_), 156.8 (s, C_ipso_), 155.9 (s, CH_cytosine_), 149.1 (s, CH_py_), 138.2 (s, CH_py_), 123.6 (s, CH_py_), 123.6 (s, CH_py_), 99.9 (s, CH_cytosine_), 68.8 (s, CH_cy_), 59.2 (s, CH_2_), 50.8 (s, CH_2_), 34.6 (s, CH_2,cy_), 32.8 (s, CH_2,cy_), 30.2 (s, CH_2,cy_), 26.1 (s, CH_2,cy_), 25.9 (s, CH_2,cy_), 25.0 (s, CH_2,cy_). Elemental analysis (%): C_23_H_33_AuN_6_S requires: C 44.37, H 5.34, N 13.50, S 5.15; found: C 44.52, H 5.28, N 13.71, S 4.99. HRMS (ESI-QTOF) *m*/*z* (%): calculated for C_23_H_34_AuN_6_S, 623.2226; found, 623.2260 [M + H]^+^. IR: ν (C=N): 1572 cm^–1^, ν (NH): 2987 and 2901 cm^–1^, ν (Au–S): 404 cm^–1^.

**3b**: Yield = 61%. ^1^H NMR (CD_2_Cl_2_, 400 MHz) δ: 8.59 (s, NH), 8.51 (d, CH_Ar,2_), 8.17 (d, CH_Ar,2′_), 7.75 (t, CH_Ar,4_), 7.50 (t, CH_Ar,4′_), 7.38 (d, CH_Ar,5_), 7.28 (d, CH_Ar,5′_), 7.17 (d, CH_Ar,5′_), 6.77 (t, CH_Ar,5_), 5.00–4.89 (m, H_cy_), 4.44 (s, CH_2_), 4.21–4.17 (m, H_cy_), 2.03–1.15 (m, CH_2,cy_). ^13^C{^1^H}-APT NMR (CD_2_Cl_2_, 101 MHz) δ: 168.6 (s, C_ipso,py_), 156.7 (s, C_ipso,py_), 149.0 (s, CH_py_), 138.4 (s, CH_py_), 135.0 (s, CH_py_), 127.2 (s, CH_py_), 123.7 (s, CH_py_), 123.6 (s, CH_py_), 117.8 (s, CH_py_), 68.8 (s, CH_cy_), 59.1 (s, CH_cy_), 50.9 (s, CH_2_), 34.5 (s, CH_2,cy_), 32.8 (s, CH_2,cy_), 36.2 (s, CH_2,cy_), 25.9 (s, CH_2,cy_), 25.0 (s, CH_2,cy_). Elemental analysis (%): C_24_H_33_AuN_4_S requires: C 47.52, H 5.48, N 9.24, S 5.29; found: C 47.39, H 5.61, N 9.36, S 5.34. HRMS (ESI-QTOF) *m*/*z* (%): Calculated for C_24_H_34_AuN_4_S, 607.2164; found, 607.2175 [M + H]^+^. IR: ν (C=N): 1571 cm^–1^, ν (NH): 2925 cm^–1^, ν (Au–S): 402 cm^–1^.

**3c**: Yield = 58%. ^1^H NMR (CD_2_Cl_2_, 400 MHz) δ: 9.66 (s, NH), 8.87 (s, NH), 8.55 (d, CH_Ar,2_), 7.76 (t, CH_Ar,4_), 7.69 (d, CH_Ar,2′_), 7.31 (t, CH_Ar,5_), 7.29 (d, CH_Ar,3_), 5.95 (d, CH_Ar,3′_), 4.87–4.76 (m, H_cy_), 4.44 (s, CH_2_), 4.16–4.05 (m, H_cy_), 2.00–1.04 (m, CH_2_,_cy_). ^13^C{^1^H}-APT NMR (CD_2_Cl_2_, 101 MHz) δ: 155.4 (s, CH_thiouracil,3′_), 149.0 (s, CH_py_), 138.4 (s, CH_py_), 123.9 (s, CH_py_), 123.9 (s, CH_py_), 123.8 (s, CH_py_), 123.8 (s, CH_py_), 110.0 (s, CH_thiouracil,2′_), 69.4 (s, CH_cy_), 59.7 (s, CH_cy_), 51.0 (s, CH_2_), 34.6 (s, CH_2,cy_), 32.9 (s, CH_2,cy_), 26.2 (s, CH_2,cy_), 25.9 (s, CH_2,cy_), 25.0 (s, CH_2,cy_). Elemental analysis (%): C_23_H_32_AuN_5_OS requires: C 44.30, H 5.17, N 11.23, S 5.14; found: C 44.39, H 5.46, N 11.32, S 4.98. HRMS (ESI-QTOF) *m*/*z* (%): Calculated for C_23_H_33_AuN_5_OS, 624.2066; found, 624.2066 [M]^+^. IR: ν (C=N): 1662 cm^–1^, ν (NH): 2925 cm^–1^, ν (Au–S): 404 cm^–1^.

**3d**: Yield = 68%. ^1^H NMR (CD_2_Cl_2_, 400 MHz) δ: 8.59–8.50 (m, CH_py,2_ + NH), 7.74 (td, CH_py,4_), 7.36 (d, CH_py,5_), 7.28 (td, CH_py,3_), 5.13–4.93 (m, CH_thioglucose_), 4.88–4.80 (m, CH_cy_), 4.41 (s, CH_2_), 4.18–4.11 (m, CH_cy_), 4.101–4.04 (m, CH_2,thioglucose_), 3.72–3.67 (m, CH_thioglucose_), 2.0 (s, CH_3_), 2.0 (s, CH_3_), 1.9 (s, CH_3_), 1.9 (s, CH_3_), 1.78–1.20 (m, CH_2_,_cy_). ^13^C{^1^H}-APT NMR (CD_2_Cl_2_, 101 MHz) δ: 202.5 (s, C_carbene_), 170.9 (s, C_C=O_), 170.7 (s, C_C=O_), 169.9 (s, C_C=O_), 169.8 (s, C_C=O_), 156.6 (s, C_ipso,py_), 149.0 (s, CH_py_), 138.2 (s, CH_py_), 123.5 (s, CH_py_), 123.7 (s, CH_py_), 83.8 (s, CH_thioglucose_), 78.1 (s, CH_thioglucose_), 75.9 (s, CH_thioglucose_), 69.7 (s, CH_py_), 68.6 (s, CH_thioglucose_), 63.3 (s, CH_2,thioglucose_), 58.9 (s, CH_py_), 50.8 (s, CH_thioglucose_), 34.5 (s, CH_2,cy_), 26.4 (s, CH_2,cy_), 26.2 (s, CH_2,cy_), 25.9 (s, CH_2,cy_), 25.8 (s, CH_2,cy_), 25.1 (s, CH_2,cy_), 24.9 (s, CH_2,cy_), 21.5 (s, CH_3_), 21.1 (s, CH_3_), 21.0 (s, CH_3_), 20.9 (s, CH_3_). Elemental analysis (%): C_33_H_48_AuN_3_O_9_S requires: C 46.10, H 5.63, N 4.89, S 3.73; found: C 46.33, H 5.52, N 4.77, S 3.67. HRMS (ESI-QTOF) *m*/*z* (%): Calculated for C_33_H_48_AuN_3_O_9_SNa, 882.2669; found, 882.2761 [M + H]^+^. IR: ν (C=N): 1557 cm^–1^, ν (NH): 2923 cm^–1^, ν (Au–S) 405 cm^–1^.

### Cytotoxicity Assay

The MTT assay was used to determine cell viability as an indicator for cell sensitivity to the complexes. Exponentially growing cells A549, HCT116 WT, HCT DKO Jurkat, MiaPaca2, and healthy lymphocytes T were seeded at a density of approximately 1 × 10^4^ cells per well in 96-well flat-bottomed microplates. The complexes were dissolved in DMSO and then diluted with culture medium to obtain the final concentrations ranging from 0.1 to 50 μM in quadruplicate. Cells were incubated with the compounds for 24 h at 37 °C. 10 μL of MTT (5 mg mL^–1^) was added to each well and plates were incubated for 2 h at 37 °C. Then, media were discarded and DMSO (100 μL per well) was added to dissolve the formazan precipitates in the plates. Plates containing Jurkat were previously centrifuged for 15 min at 2500 rpm. Thereafter, media were also eliminated, and DMSO (100 μL per well) was added. The optical density was measured at 490 nm using a 96-well multiscanner autoreader, UV–visible ELISA. The IC_50_ value was calculated by nonlinear regression analysis using OriginPro.

#### Cell Death Study

Cell death was analyzed by measuring the translocation of phosphatidylserine from the inner to outer cell membrane. Cells Jurkat and Jurkat shBak were titrated with compounds **2a** and **3d** for 24 h at 37 °C. Then, they were trypsinized and incubated at 37 °C for 15 min in ABB (140 mM NaCl, 2.5 mM CaCl_2_, 10 mM Hepes/NaOH, pH 7.4) containing 0.5 mg mL^–1^ of annexin V-DY634. Finally, cells were diluted to 0.5 mL with ABB and analyzed by flow cytometry (FACSCalibur, BD Biosciences, Spain).

#### DNA Binding

In DNA-binding experiments, the complexes were dissolved in DMSO and diluted with the Tris–HCl buffer (10 mM, pH = 7.2) with a final concentration of DMSO of 20%. The absorption spectra were performed in fixed concentration of metal complexes (100 μM) while gradually increasing the concentration of CT-DNA from 0 to 100 μM. To obtain the absorption spectra, the required amount of CT-DNA was added to both compound solution and the reference solution to eliminate the absorbance of CT-DNA itself. Each sample solution was allowed to equilibrate 10 min before the spectra were recorded. Using the absorption titration data, the binding constant K_b_ was determined using the Wolfe–Shimer equation:^48^
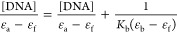
where [DNA] is the concentration of CT-DNA, ε_a_ corresponds to the extinction coefficient observed (*A*_obsd_/[M]), ε_f_ corresponds to the coefficient of the free compound, ε_b_ is the extinction coefficient of the compound fully bound to CT-DNA, and *K*_b_ is the intrinsic binding constant. The *K*_b_ value was determined by the ratio of the slope to the intercept in the plot of [DNA]/(ε_a_ – ε_f_) versus [DNA].

#### Thioredoxin Reductase Inhibition Assay in A549 Cells

The thioredoxin reductase inhibition assay for the compounds was tested on A549 cells using a method reported by Holmgren et al.^46^ Briefly, cells were seeded in 24-well plates at a density of 1 × 10^5^ cells/well in DMEM/10% FBS. After 48 h, they were treated with 3 μM of the compounds (**2b**, **3b**, **3d**, and auranofin) dissolved in DMSO (0.1%) and one control (only 0.1% DMSO) and incubated at 37 °C, 5% CO_2_ for 24 h. Following this, the media were removed, and cells were washed twice with cold PBS and lysed with ice-cold lysis buffer (50 mM Phosphate buffer pH 7.4; 1 mM EDTA, 0.1% Triton-X 100) for 15 min on ice. The protein content in the samples was estimated using the Bradford assay with bovine serum albumin (BSA) as a calibration standard. Equal amounts of protein (5 μg) were used. Samples were incubated with 30 μL of reaction mixture (HEPES buffer (0.2 mM), insulin (3 mg/mL), NADPH (1 mM), EDTA (2 mM), and either 5 μL of recombinant thioredoxin (1 mg/mL) or 5 μL of HEPES buffer (200 μM)) for 30 min at 37 °C. The reaction was stopped with 200 μL of stopping solution containing guanidine hydrochloride (5.4 M in TRIS–HCl 100 mM) and 5,5′-dithio-bis(2-nitrobenzoic acid) (1 mM). The absorbance was measured at 405 nm by using a FLUOstar Omega (BMG LABTECH) plate reader. The difference in the absorbance of samples containing Trx and buffer gave the activity of thioredoxin reductase expressed as a percentage inhibition relative to the control. The SD was calculated by using two independent experiments.

### Cell Death, Cell Cycle, ROS, and Mitochondrial Membrane Potential

200,000 cells (A549/mL) were seeded in flat-bottom 6-well plates (1 mL/well) in complete medium and allowed to attach for 24 h. A solution of the complex (**3b**) was added at a concentration of 4xIC_50_ and A549 cells were cultured for a total of 24 h. The cells were then deadhered with 200 μL of trypsin and resuspended in 1 mL of media. This cell suspension has been used for all flow cytometry measurements that have been carried out at cytometer SA3800 Sony (cell cycle) and cytometer GALLIOS Beckman Coulter. Commercial kits were used for all flow cytometry studies and in all cases, they were used as indicated in the manufacturer’s instructions. Cell death studies: ANNEXIN V FITC Apoptosis detection kit (immunostep, reference ANXVKF-100T). Cell cycle: PI/RNASE Solution 200 test (immunostep, reference PI/RNASE). ROS production: CellROX Green and CellROX Orange Flow Cytometry Assay Kits (molecular probes by life technologies, catalogue number C10492). Mitochondrial potential: MitoStep Flow Cytometry Mitochondrial Membrane Potential Assay (immunostep, reference: MITO-100T).

### Crystallography

Crystals were mounted in inert oil on glass fibers and transferred to the cold gas stream of an Xcalibur Oxford Diffraction (**1a**) or a Smart APEX CCD diffractometer (**1a′**, **2a**, **3d**) equipped with a low-temperature attachment. Data were collected using monochromated Mo-Kα radiation (λ = 0.71073 Å). Scan type ϖ. Absorption corrections based on multiple scans were applied using SADABS^49^ or spherical harmonics implemented in SCALE3 ABSPACK scaling algorithm.^50^ The structures were solved by direct methods and refined on *F*^2^ using the program SHELXT-2018.^51^ All non-hydrogen atoms were refined anisotropically.

## Supporting Information Available

Additional experimental details including NMR data, X-ray data, stability studies by UV–vis and NMR, mechanism of cell death, and other flow cytometry studies (PDF)
