# Supplementary material for: Gold Acyclic Diaminocarbene Complexes as Selective and Potent Agents for Multitarget Cancer Therapy
Source: Inorg Chem. 2025 May 5;64(19):9608–20. doi: 10.1021/acs.inorgchem.5c00579 (PMC12093293; doi:10.1021/acs.inorgchem.5c00579)
Supplement: Supplementary file 1 — ic5c00579_si_001.pdf [file ic5c00579_si_001.pdf]

SUPPORTING INFORMATION

Gold Acyclic Diaminocarbene Complexes as Selective and Potent Agents for Multitarget Cancer Therapy

María Gil-Moles,[a] Melanie Aliaga-Lavrijsen,[a] Sara Montanel-Pérez,[a] Isabel Marzo,[b] M. Dolores Villacampa,[a] M. Concepción Gimeno[a,*]

Corresponding author email: gimeno@unizar.es

[a]Departamento de Química Inorgánica, Instituto de Síntesis Química y Catálisis Homogénea (ISQCH) CSIC-Universidad de Zaragoza, 50009 Zaragoza, Spain

[b]Departamento de Bioquímica y Biología Celular, Universidad de Zaragoza, 50009 Zaragoza, Spain

Table of Contents

1	**^{1}H NMR and ^{13}C-APT NMR: Figures S1-S21**	*S2*
2	**X-Ray: Figure S22**	*S13*
3	**Stability Studies: Figures S23-S32 (NMR) Figures S33-S42**	*S13*
4	**Studies of the mechanism of cell death: Figures S43-S45**	*S23*
5	**Flow cytometry: Figures S46-S48**	*S25*

1 ^{1}H NMR and ^{13}C-APT NMR: Figures S1-S20

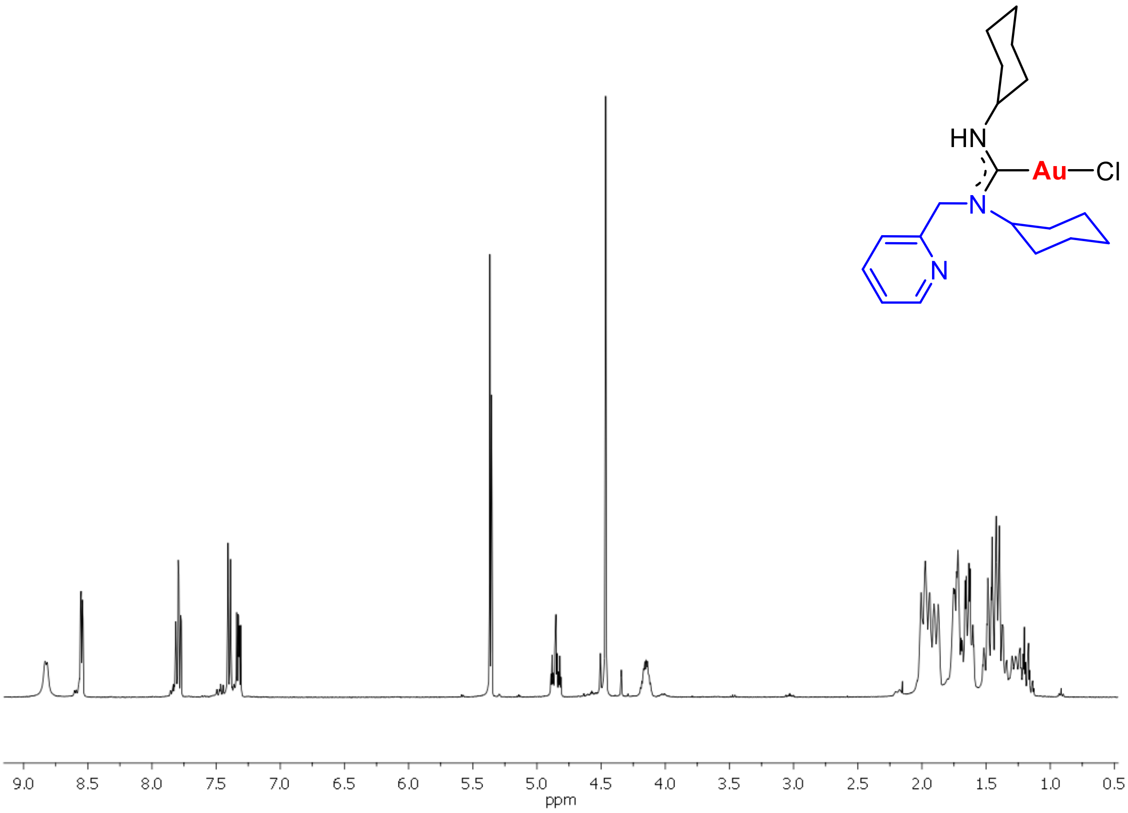

Figure S1: ^{1}H NMR (400 MHz CD_2Cl_2) of complex **1a**.

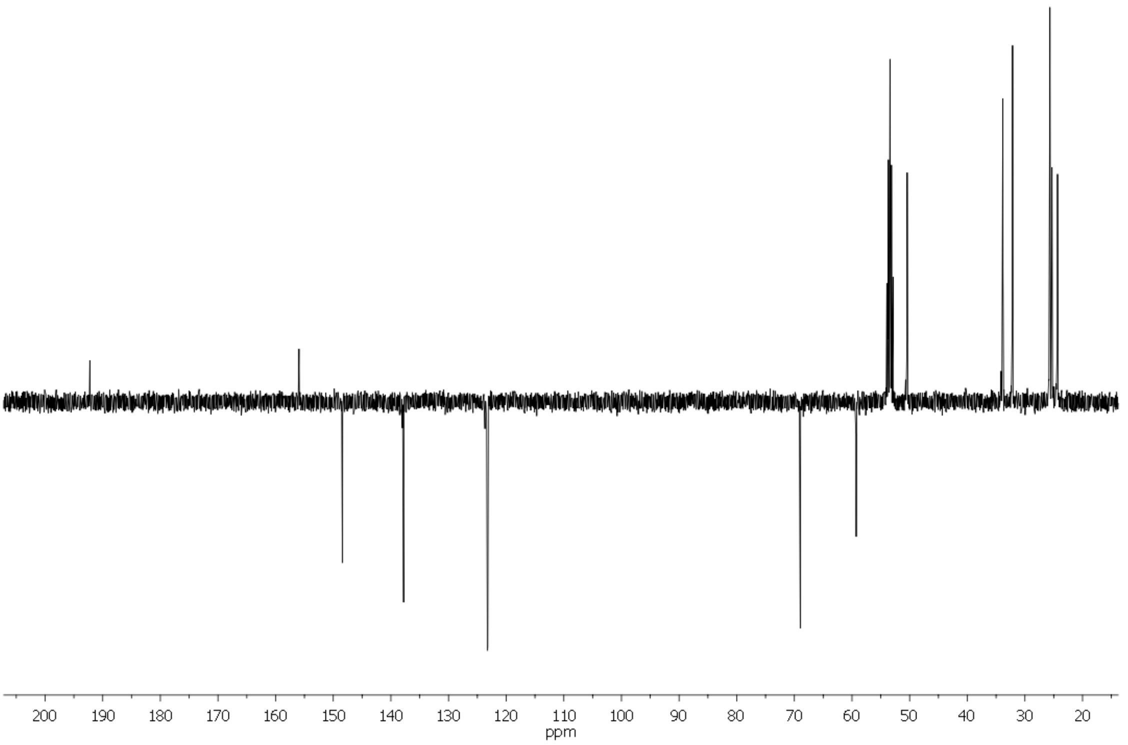

Figure S2: ^{13}C–APT NMR (101 MHz CD_2Cl_2) of complex **1a**.

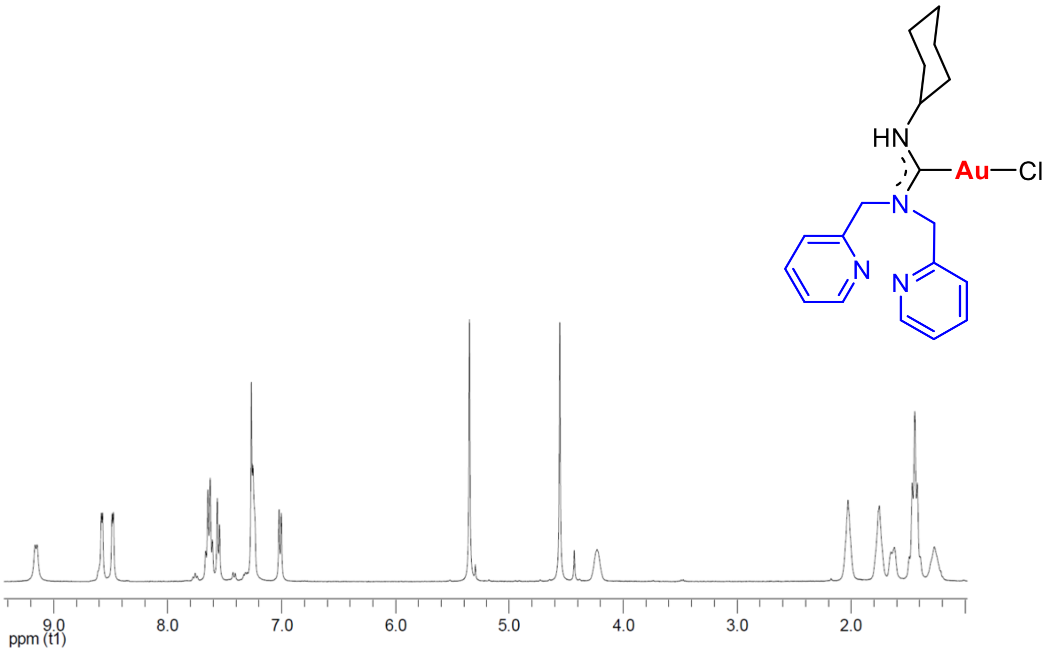

Figure S3: ^{1}H NMR (400 MHz $CDCl_3$) of complex **1b**.

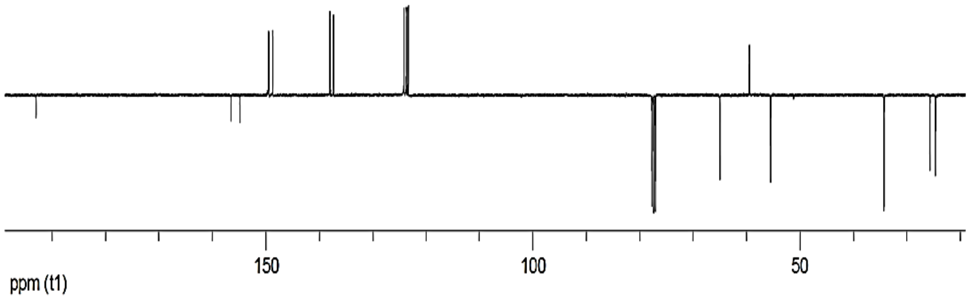

Figure S4: ^{13}C–APT NMR (101 MHz $CDCl_3$) of complex **1b**.

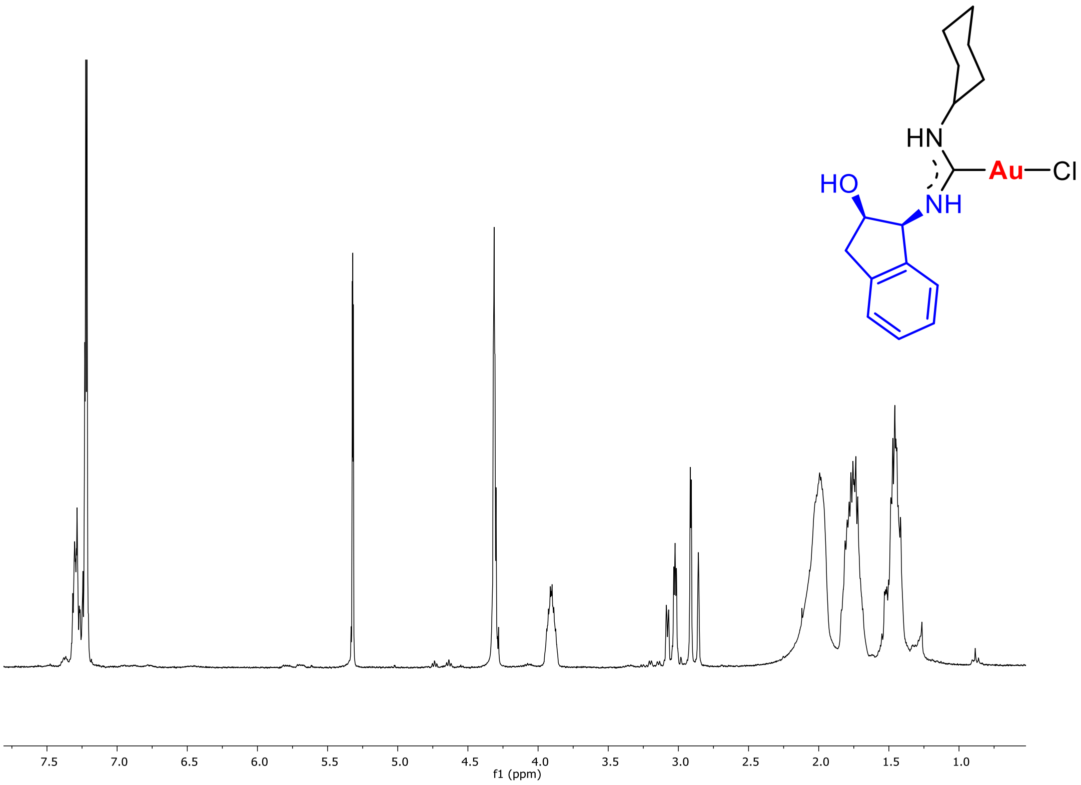

Figure S5: ^{1}H NMR (400 MHz CD_2Cl_2) of complex **1c**.

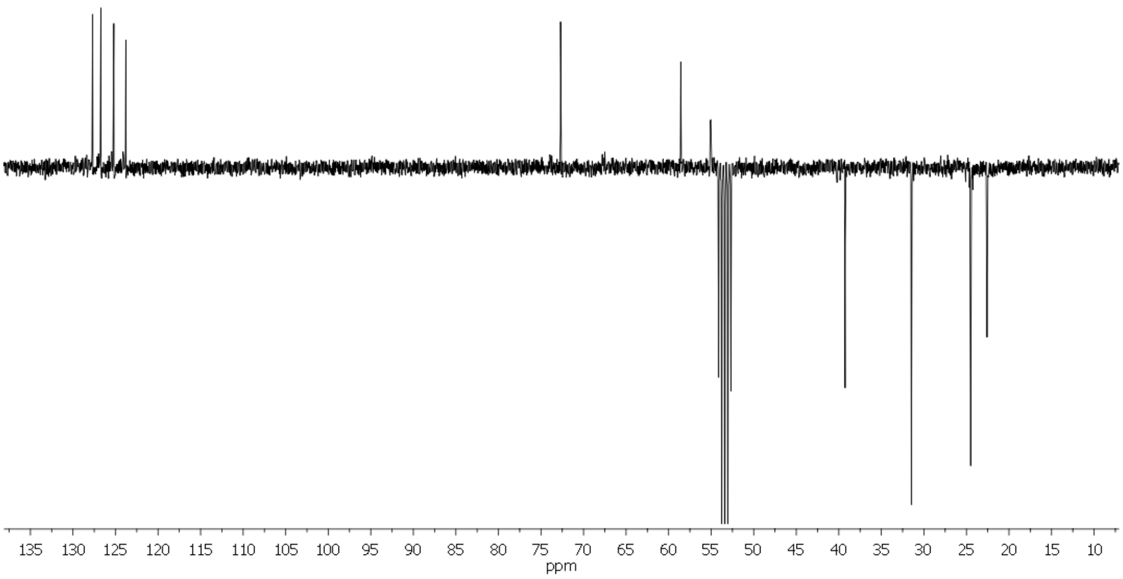

Figure S6: ^{13}C–APT NMR (101 MHz CD_2Cl_2) of complex **1c**.

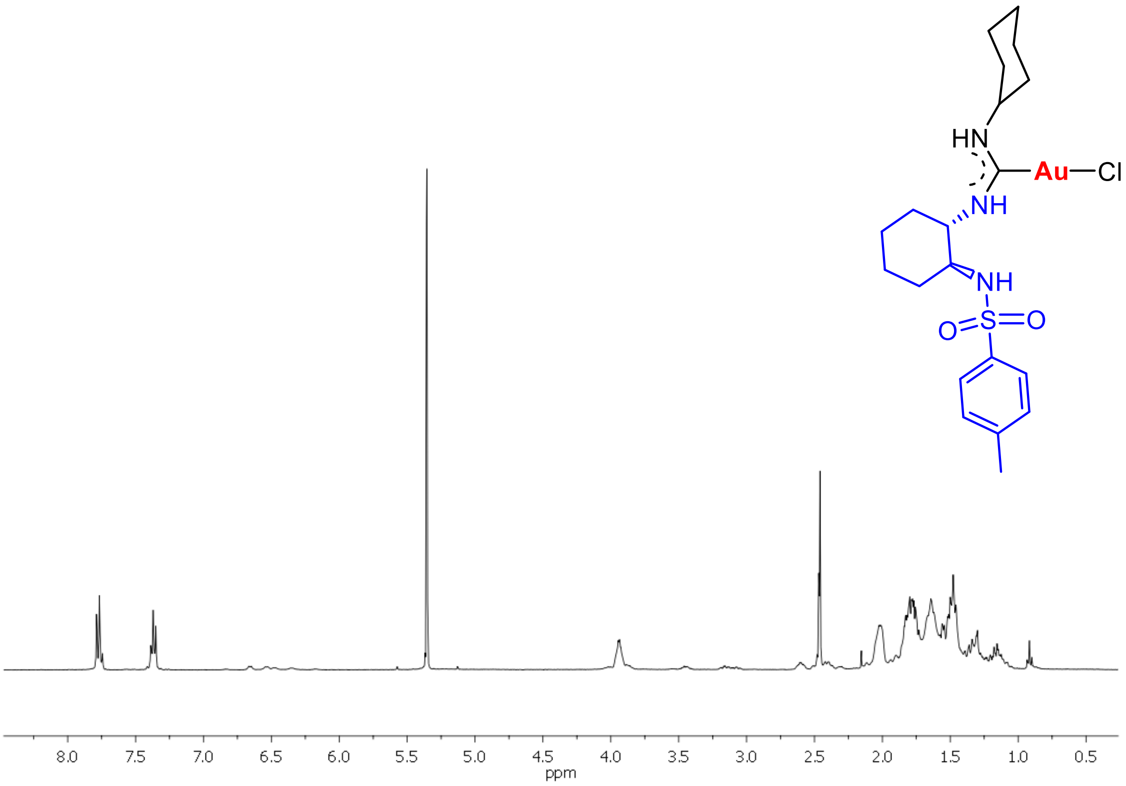

Figure S7: ^{1}H NMR (400 MHz CD_2Cl_2) of complex **1d**.

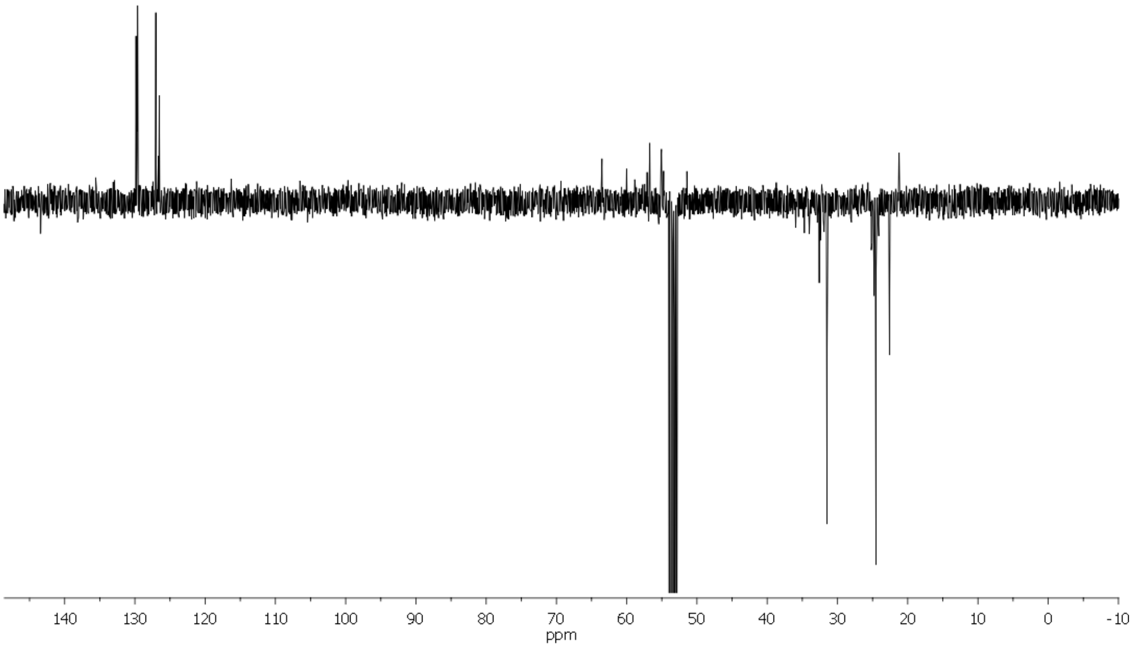

Figure S8: ^{13}C–APT NMR (101 MHz CD_2Cl_2) of complex **1d**.

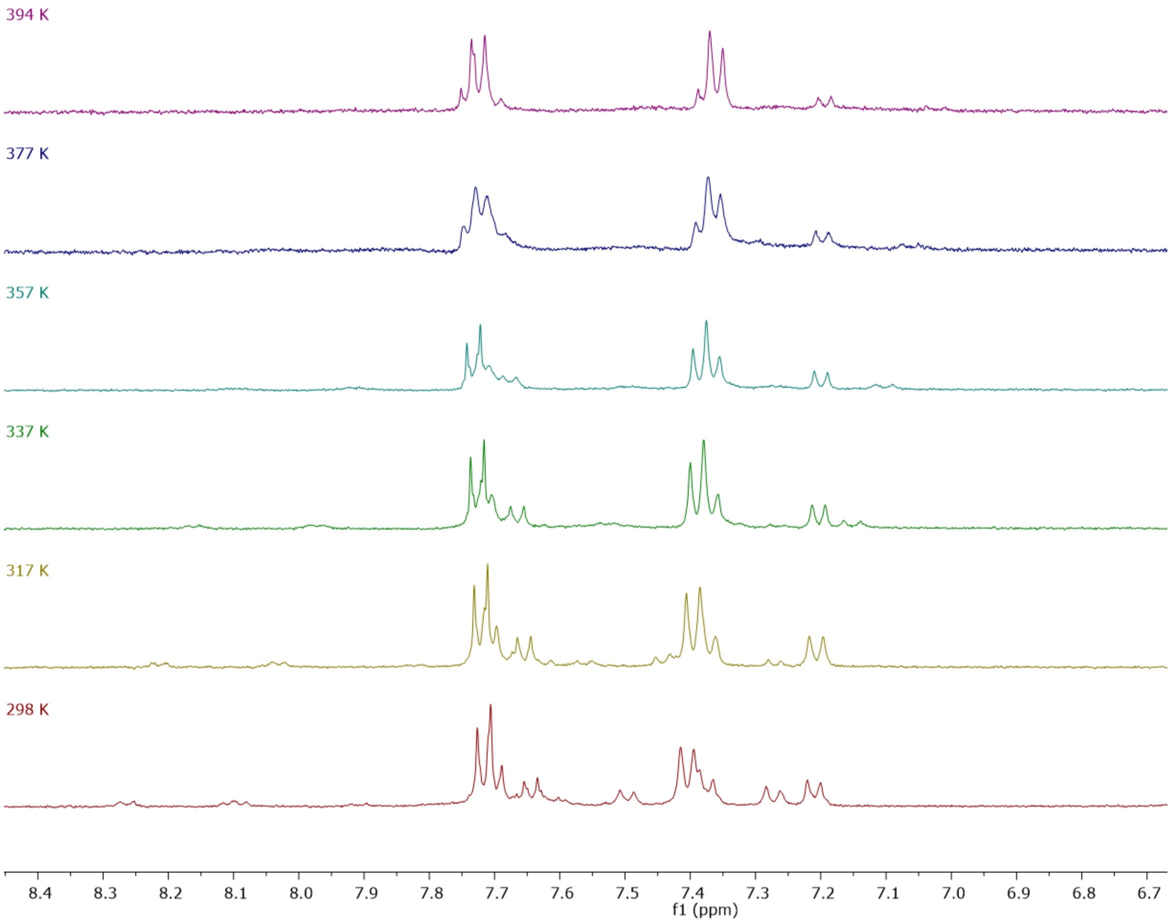

Figure S9: NMR in d_6-DMSO at different temperatures of compound **1d** (for clarity, only the aromatic region is shown).

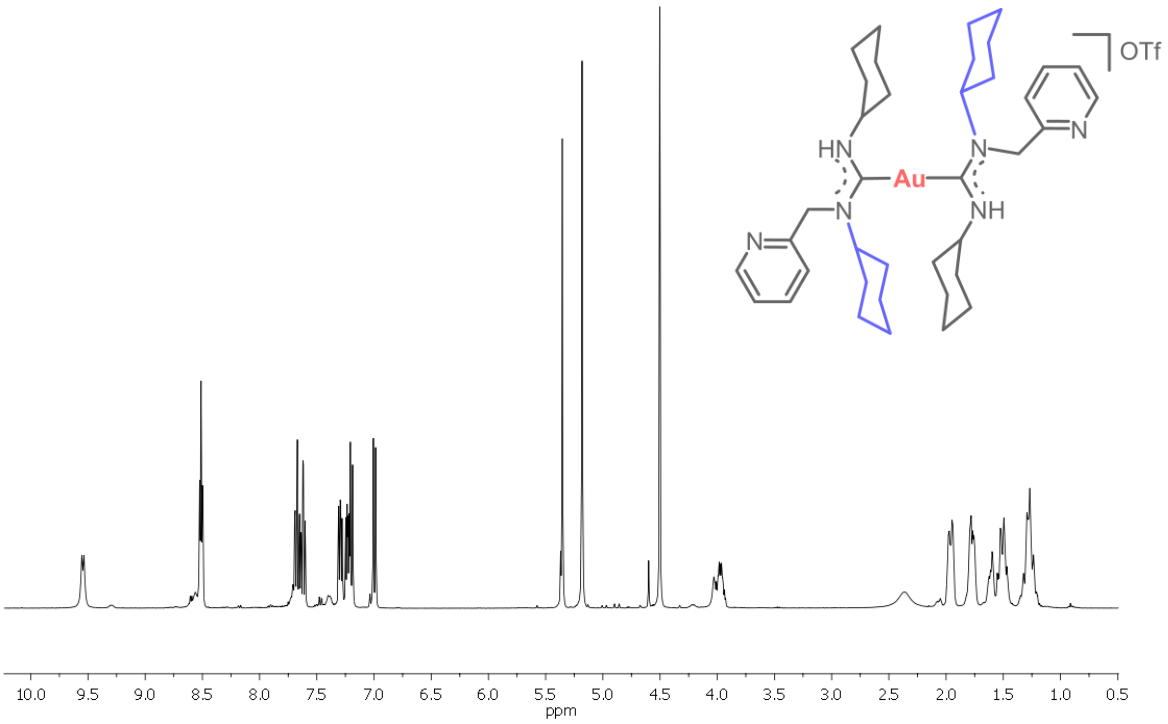

Figure S10: ^{1}H NMR (400 MHz CD_2Cl_2) of complex **2a**.

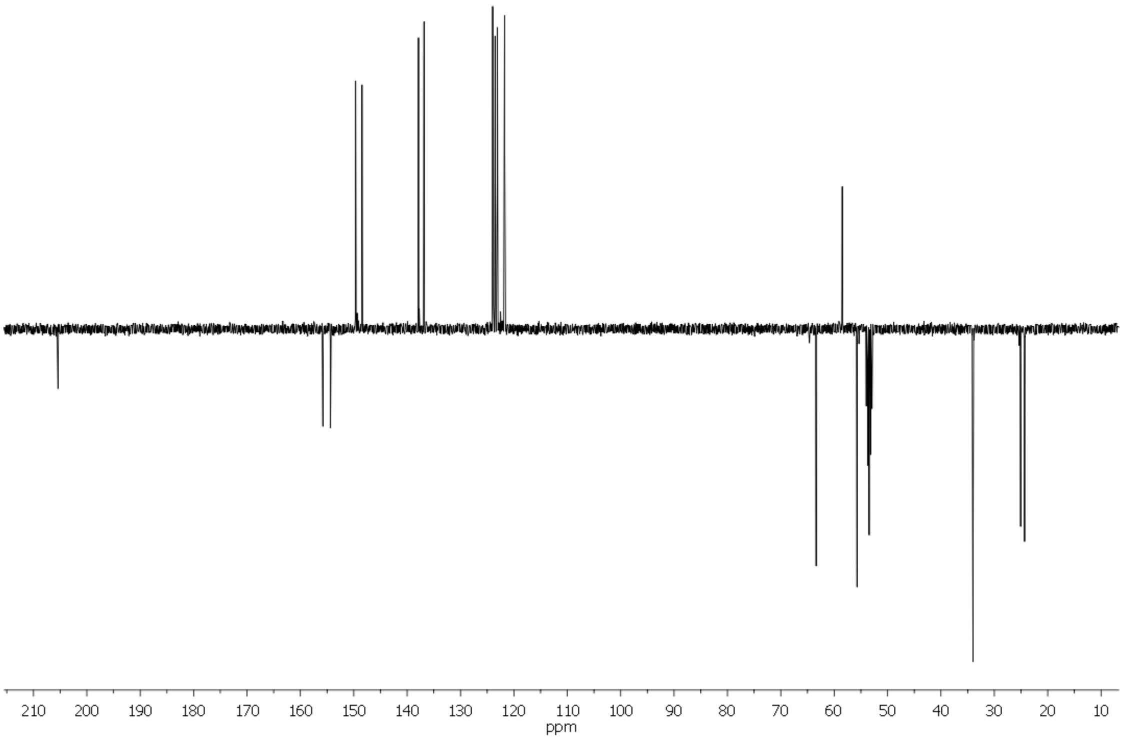

Figure S11: ^{13}C–APT NMR (101 MHz CD_2Cl_2) of complex **2a**.

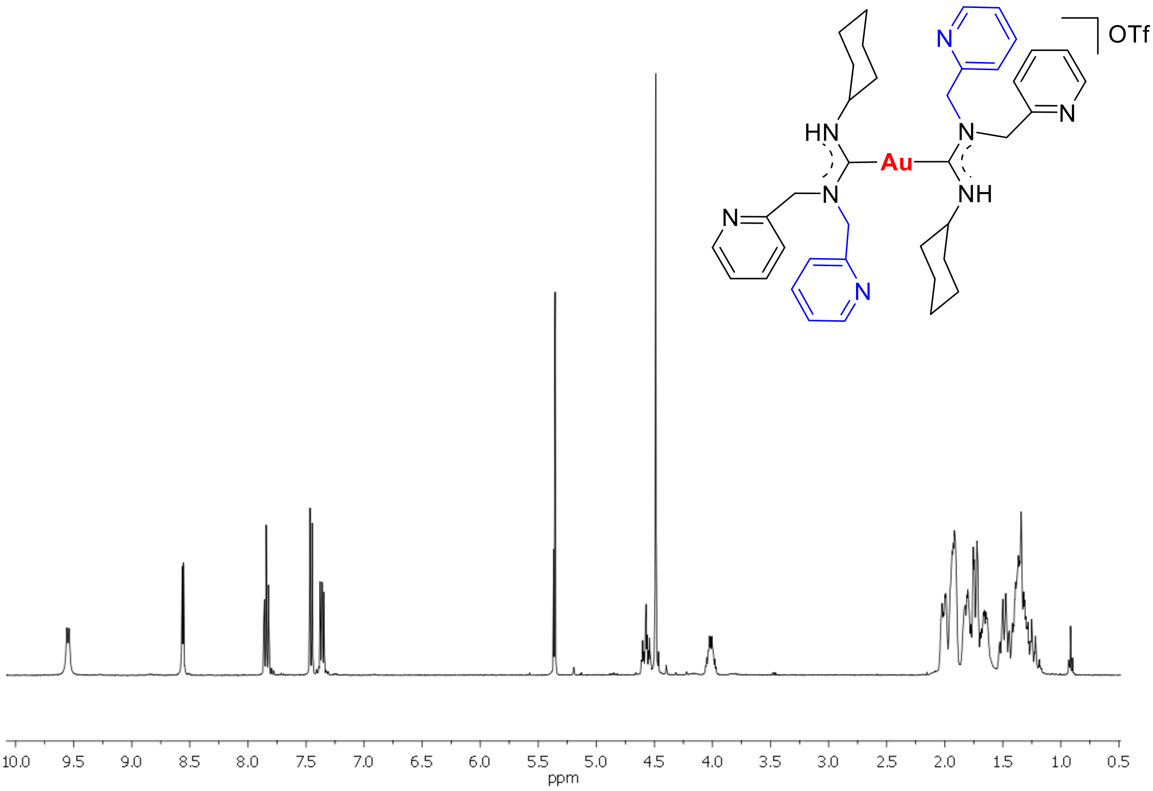

Figure S12: ^{1}H NMR (400 MHz CD_2Cl_2) of complex **2b**.

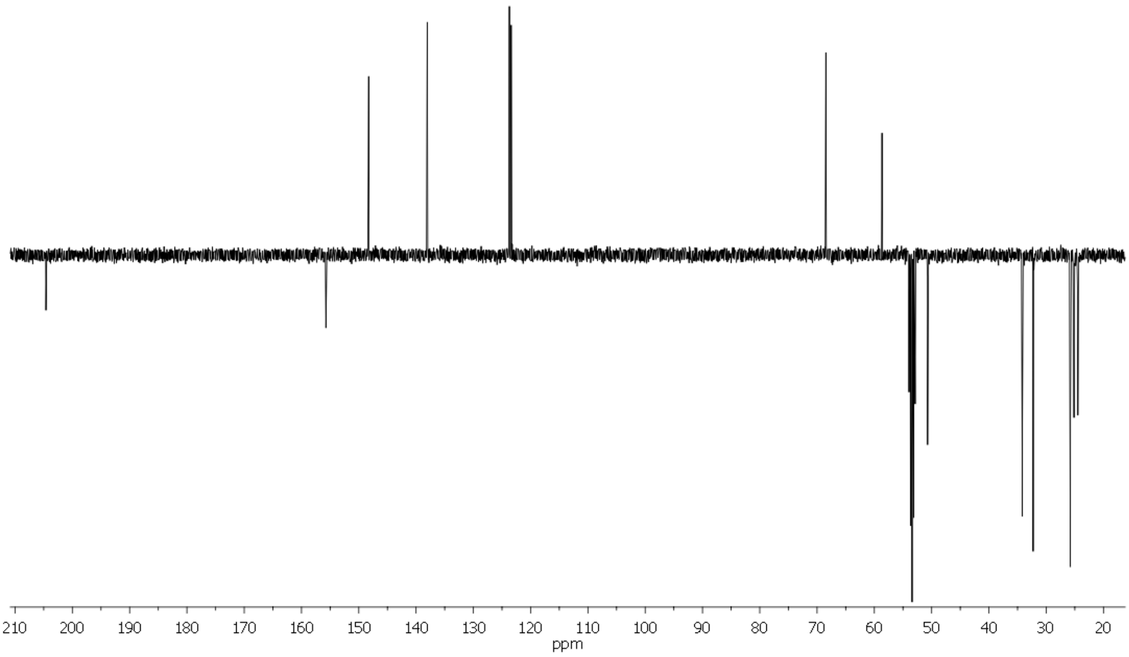

Figure S13: ^{13}C–APT NMR (101 MHz CD_2Cl_2) of complex **2b**.

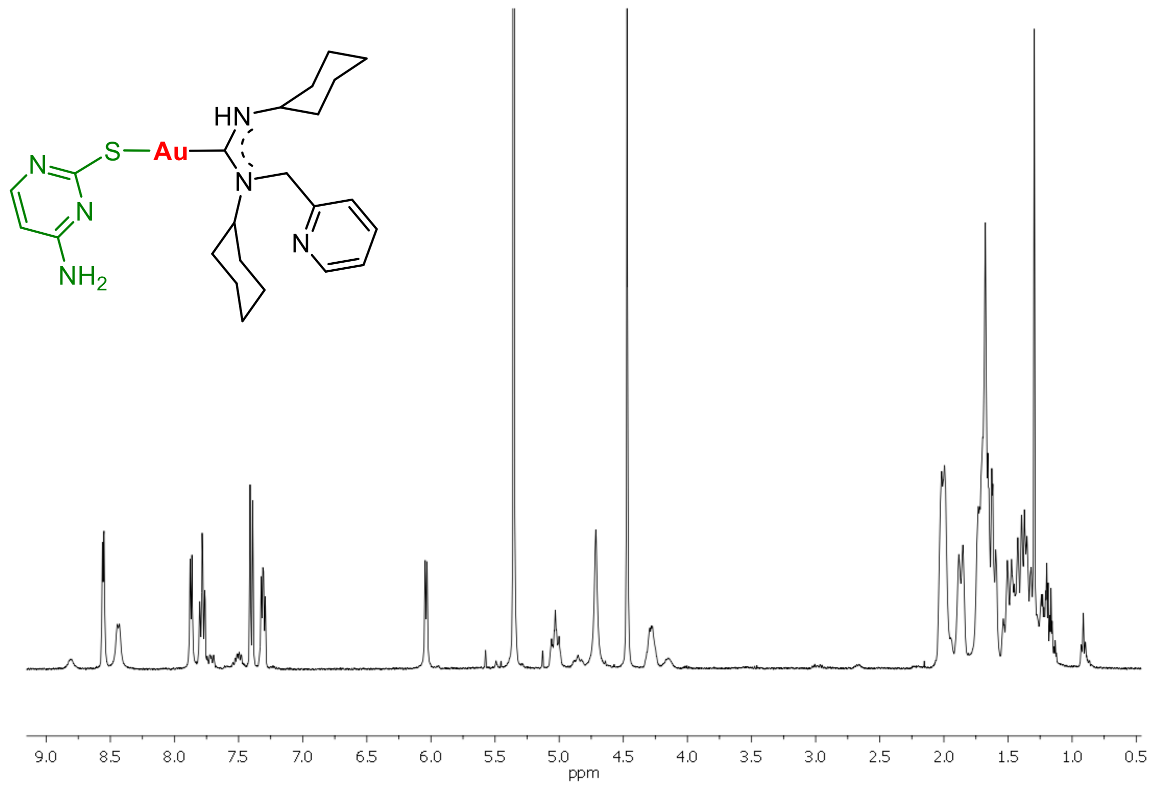

Figure S14: ^{1}H NMR (400 MHz CD_2Cl_2) of complex **3a**.

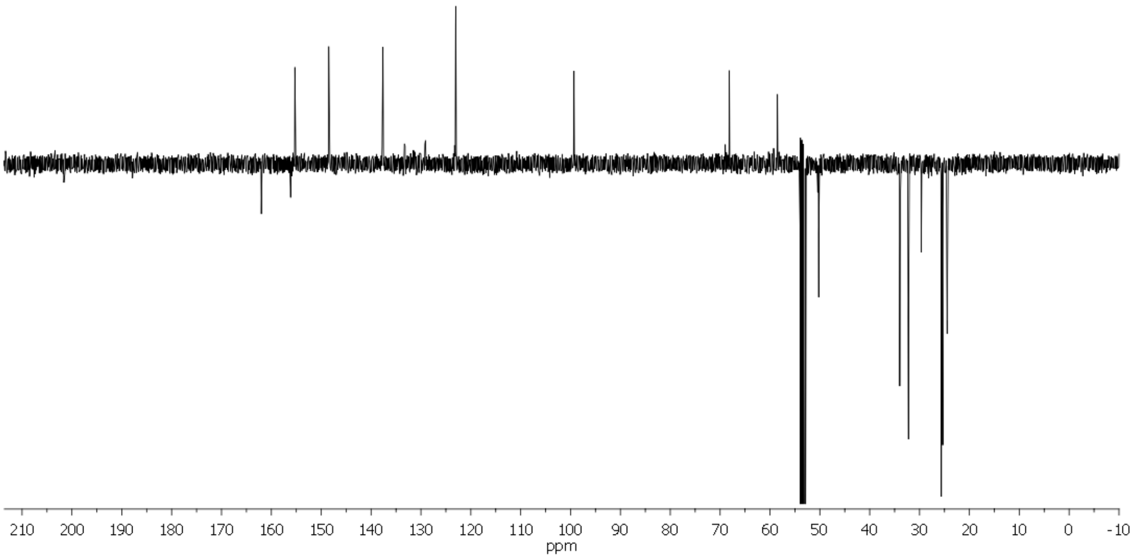

Figure S15: ^{13}C–APT NMR (101 MHz CD_2Cl_2) of complex **3a**.

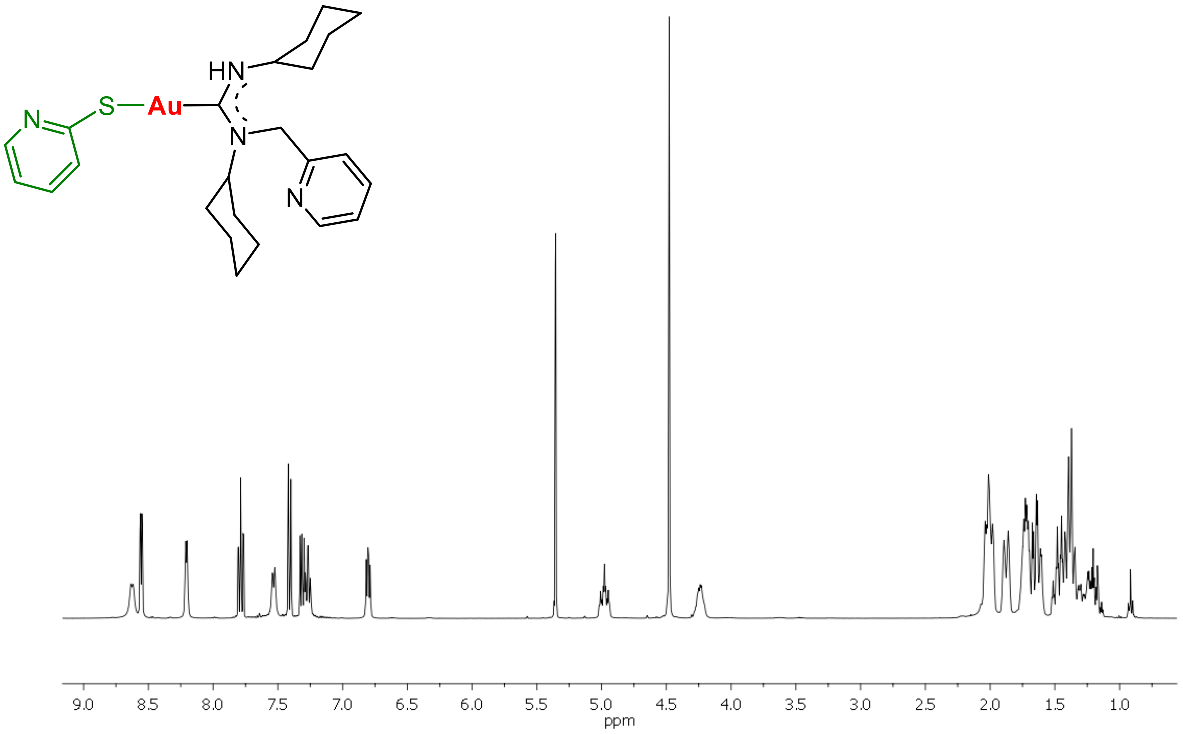

Figure S16: ^{1}H NMR (400 MHz CD_2Cl_2) of complex **3b**.

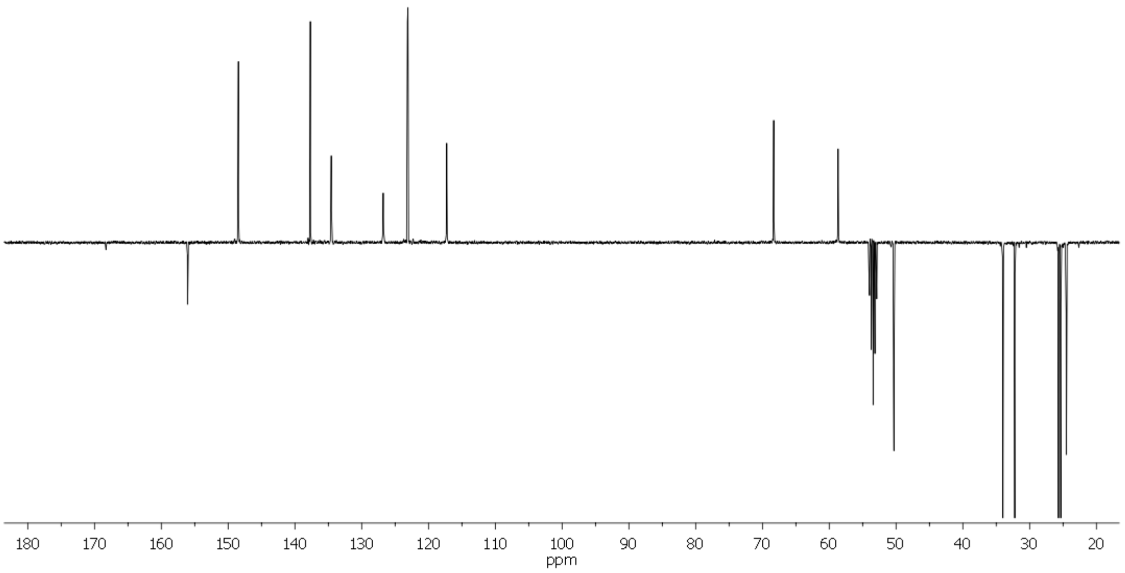

Figure S17: ^{13}C–APT NMR (101 MHz CD_2Cl_2) of complex **3b**.

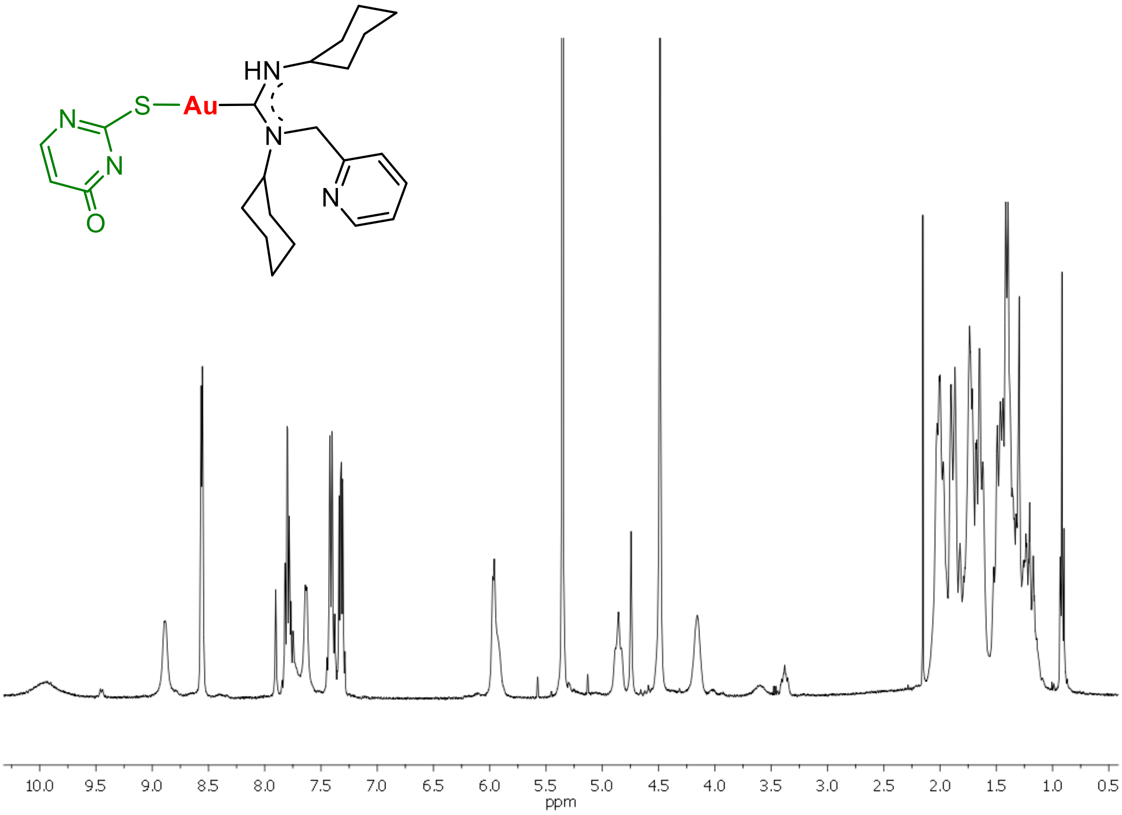

Figure S18: ^{1}H NMR (400 MHz CD_2Cl_2) of complex **3c**.

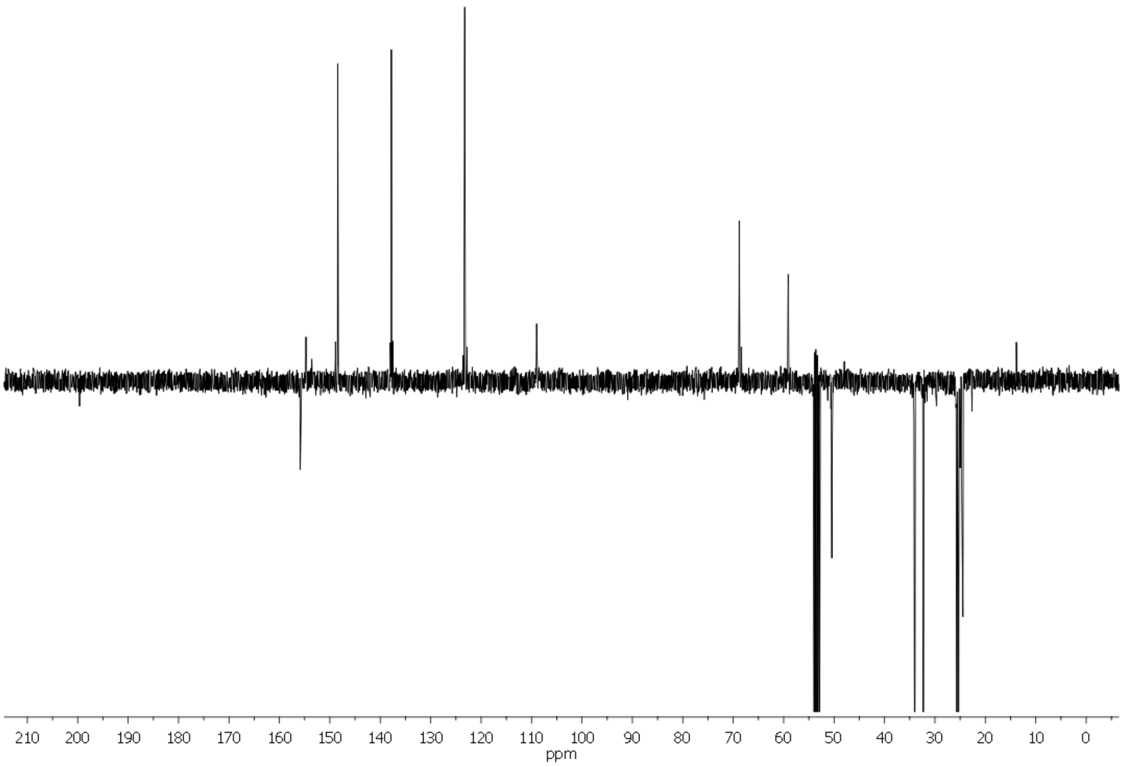

Figure S19: ^{13}C–APT NMR (101 MHz CD_2Cl_2) of complex **3c**.

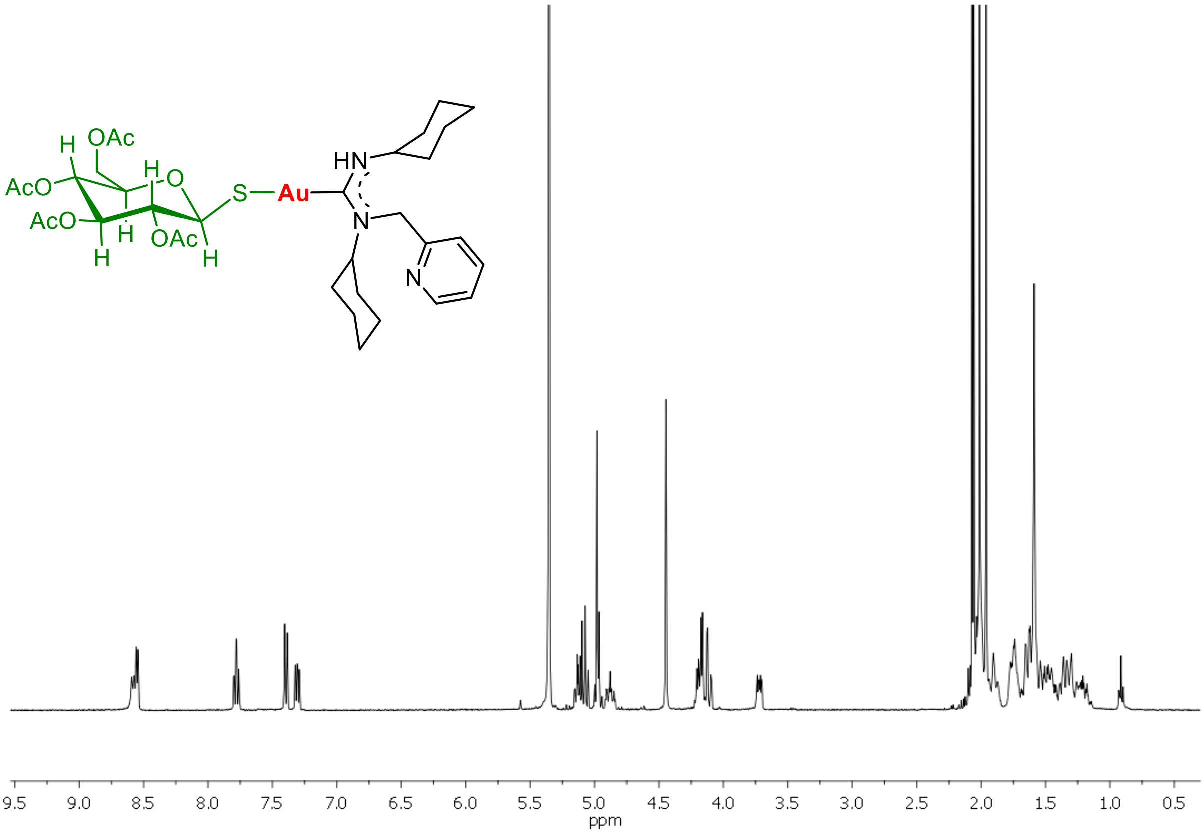

Figure S20: ^{1}H NMR (400 MHz CD_2Cl_2) of complex **3d**.

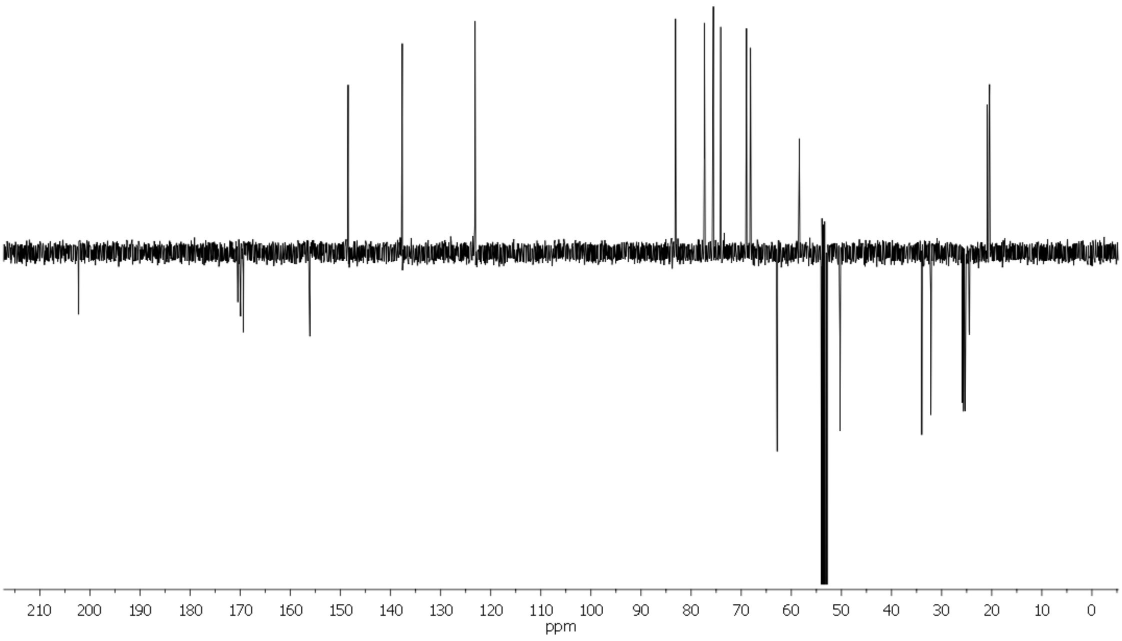

Figure S21: ^{13}C–APT NMR (101 MHz CD_2Cl_2) of complex **3d**.

2 X-Ray: Figure S22

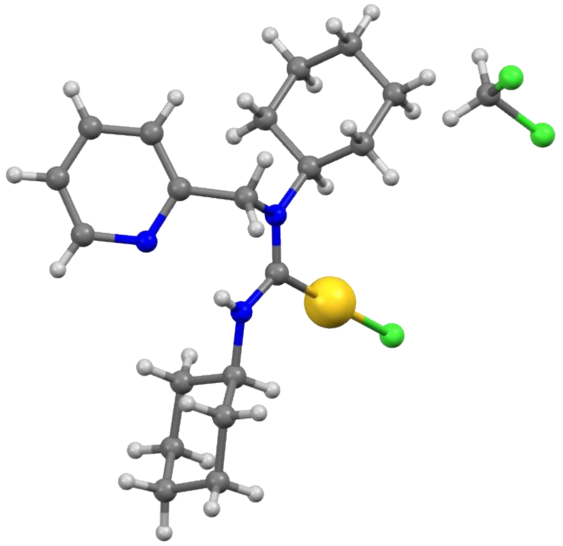

***Figure S22*:** X-Ray structure of complex 1a without aurophilic interactions

3 Stability Studies

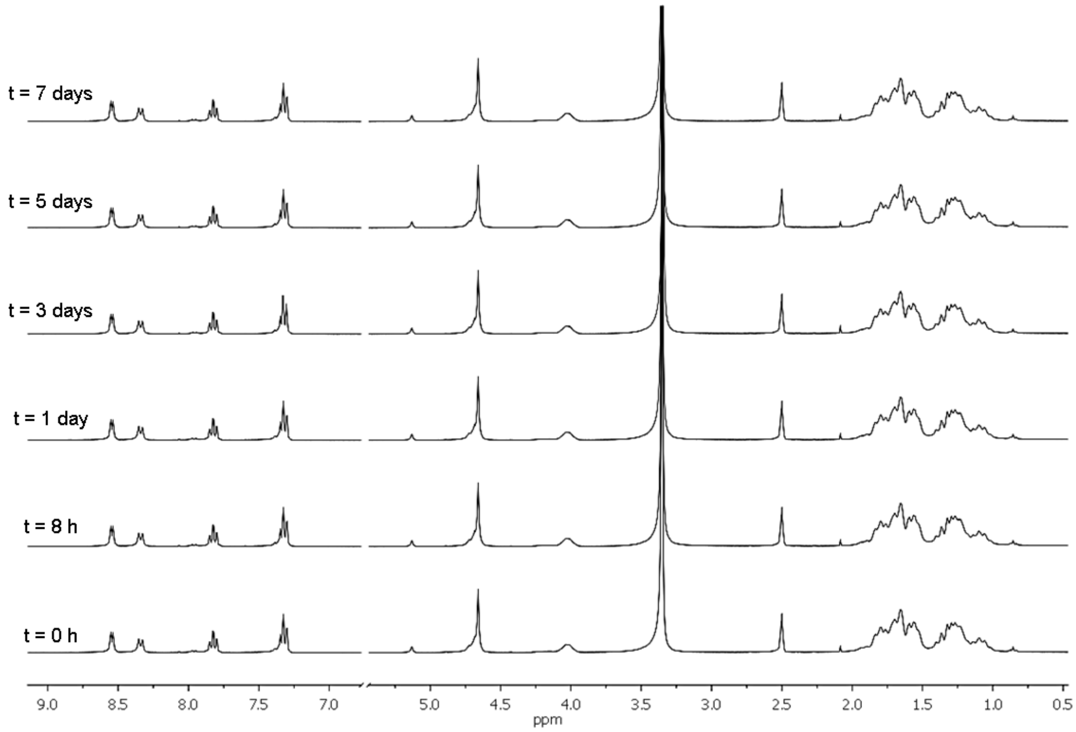

Figure S23: Stability study in ^{1}H NMR (300 MHz DMSO) of complex **1a** at different time points for 7 days.

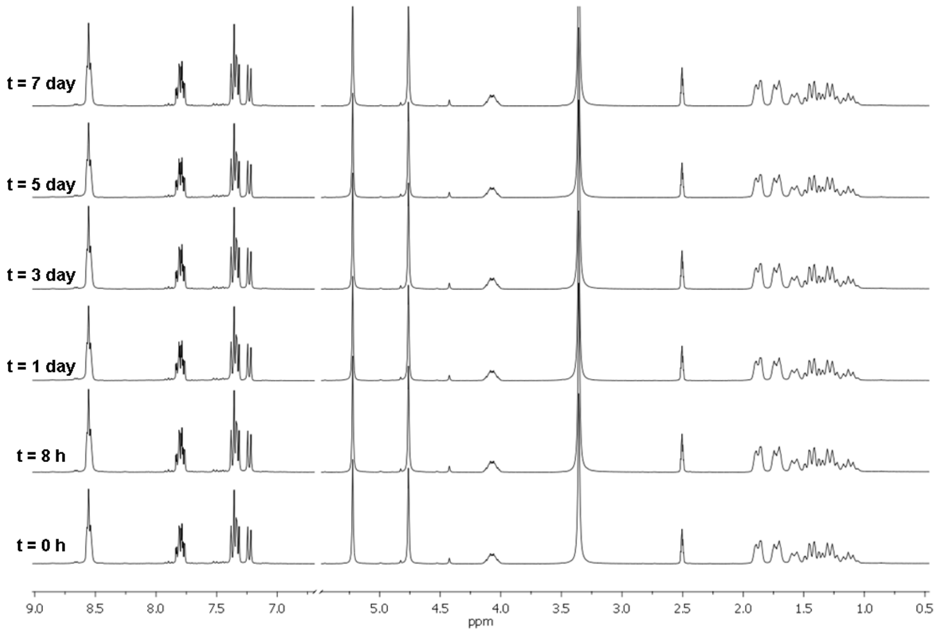

Figure S24: Stability study in ^{1}H NMR (300 MHz DMSO) of complex **1b** at different time points for 7 days.

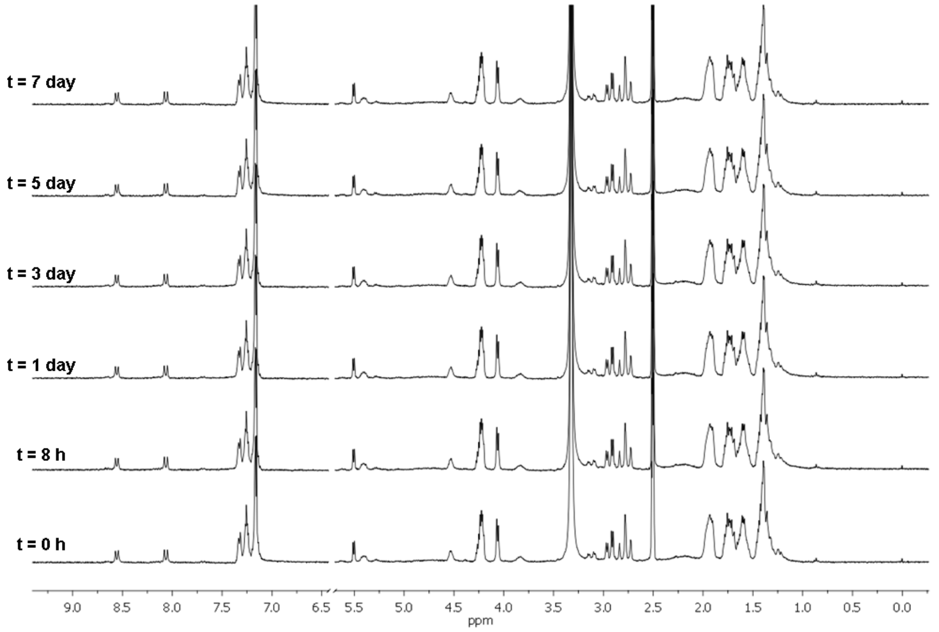

Figure S25: Stability study in ^{1}H NMR (300 MHz DMSO) of complex **1c** at different time points for 7 days.

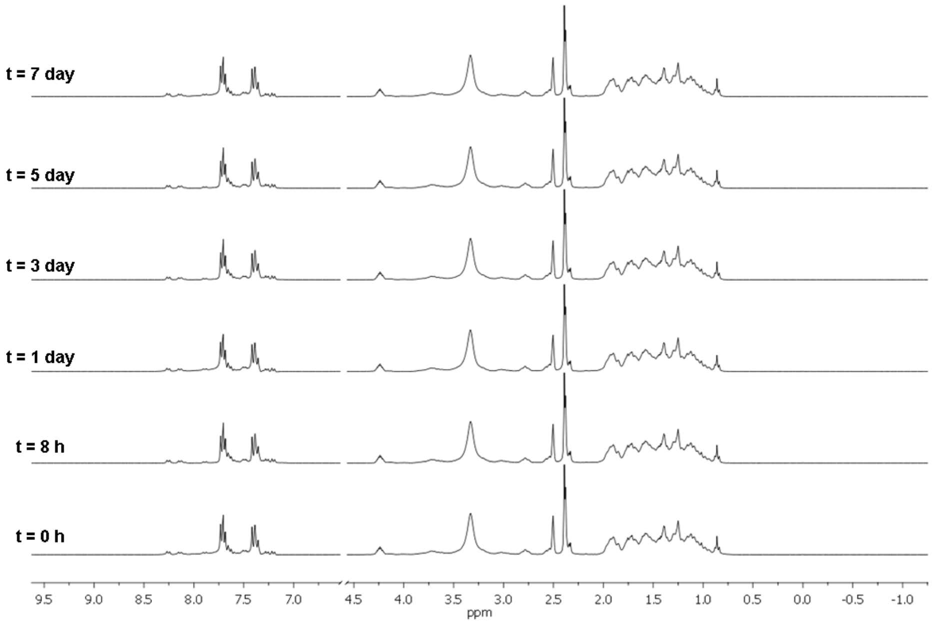

Figure S26: Stability study in ^{1}H NMR (300 MHz DMSO) of complex **1d** at different time points for 7 days.

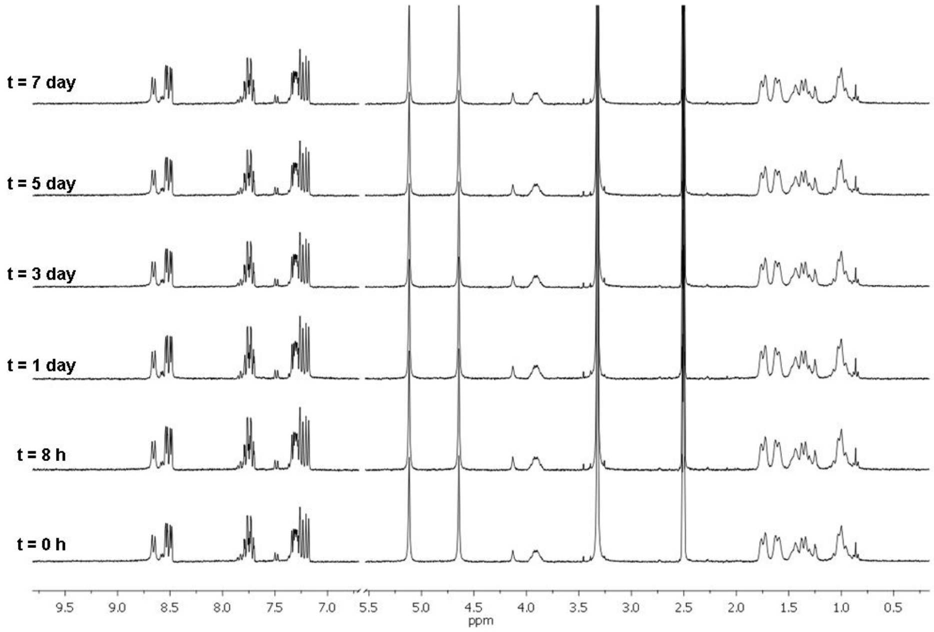

Figure S27: Stability study in ^{1}H NMR (300 MHz DMSO) of complex **2a** at different time points for 7 days.

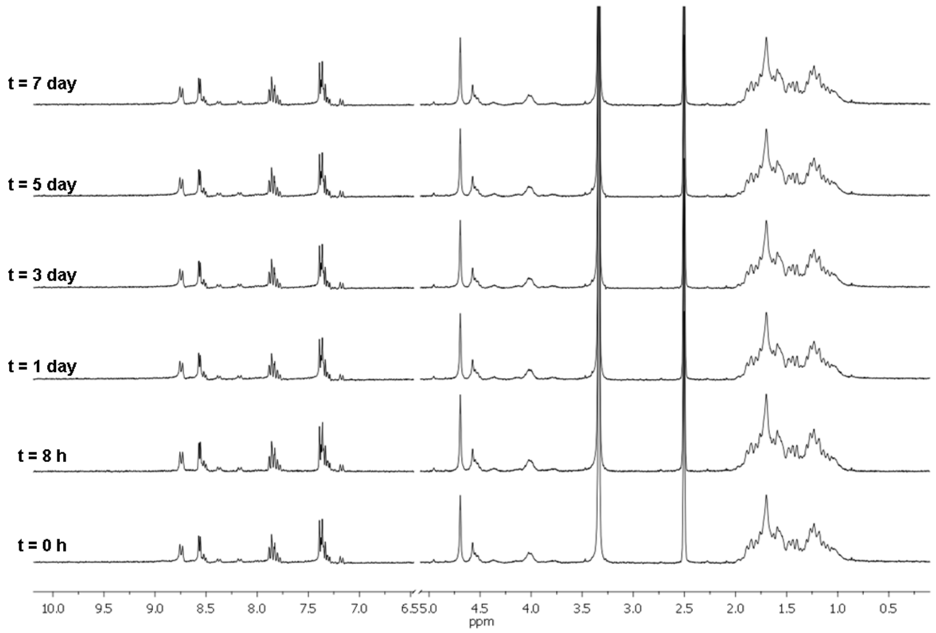

Figure S28: Stability study in ^{1}H NMR (300 MHz DMSO) of complex **2b** at different time points for 7 days.

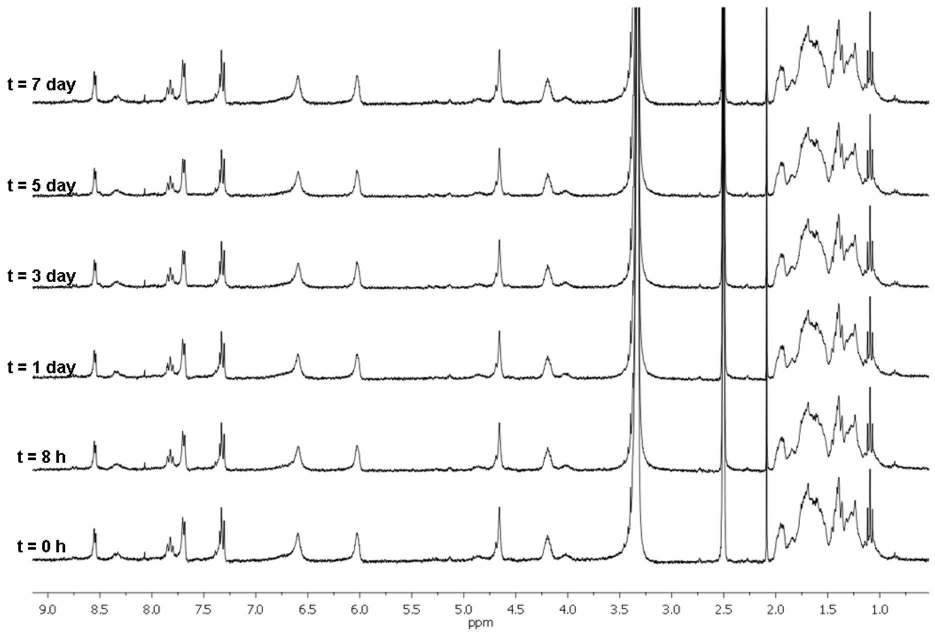

Figure S29: Stability study in ^{1}H NMR (300 MHz DMSO) of complex **3a** at different time points for 7 days.

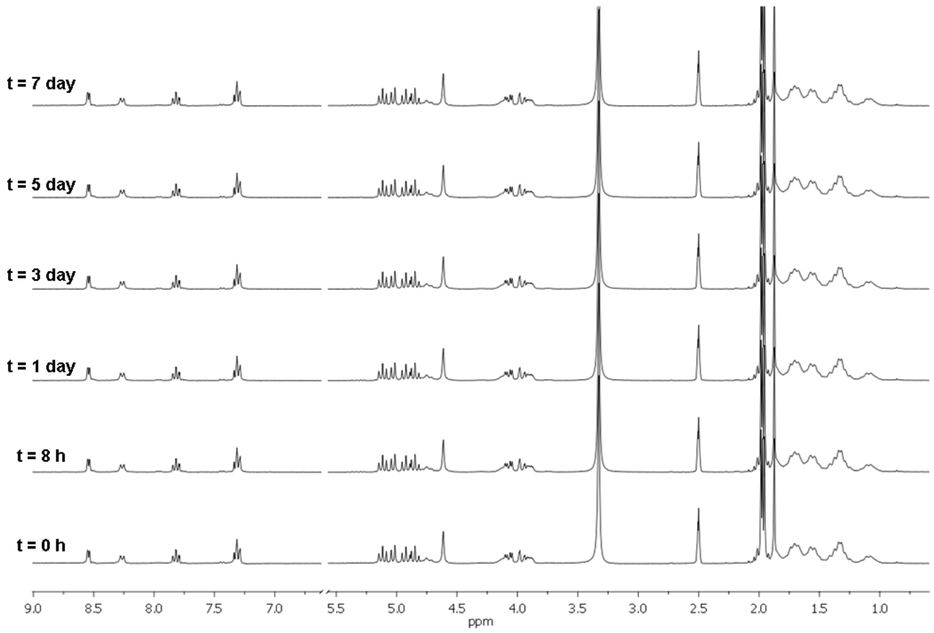

Figure S30: Stability study in ^{1}H NMR (300 MHz DMSO) of complex **3b** at different time points for 7 days.

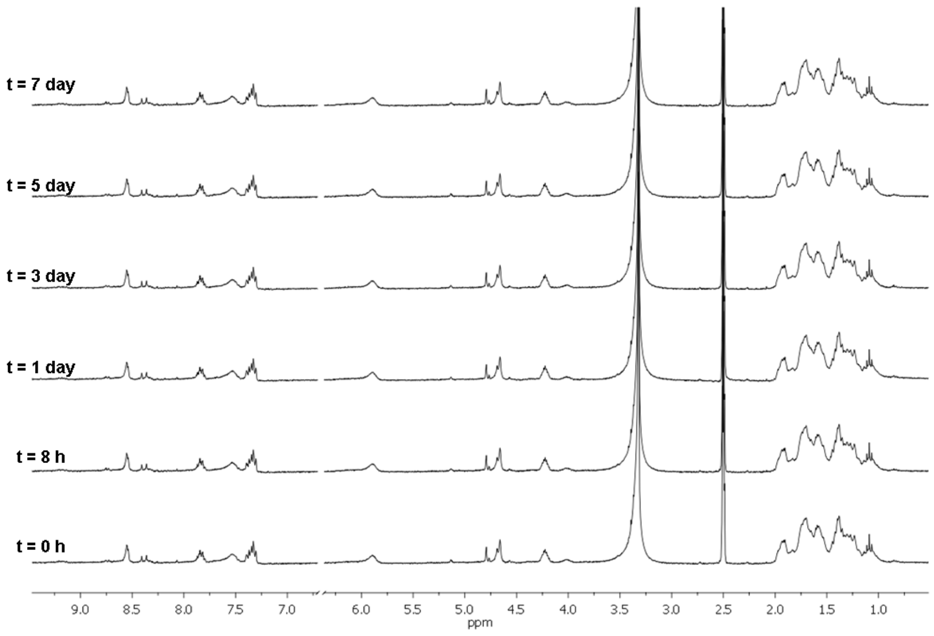

Figure S31: Stability study in ^{1}H NMR (300 MHz DMSO) of complex **3c** at different time points for 7 days.

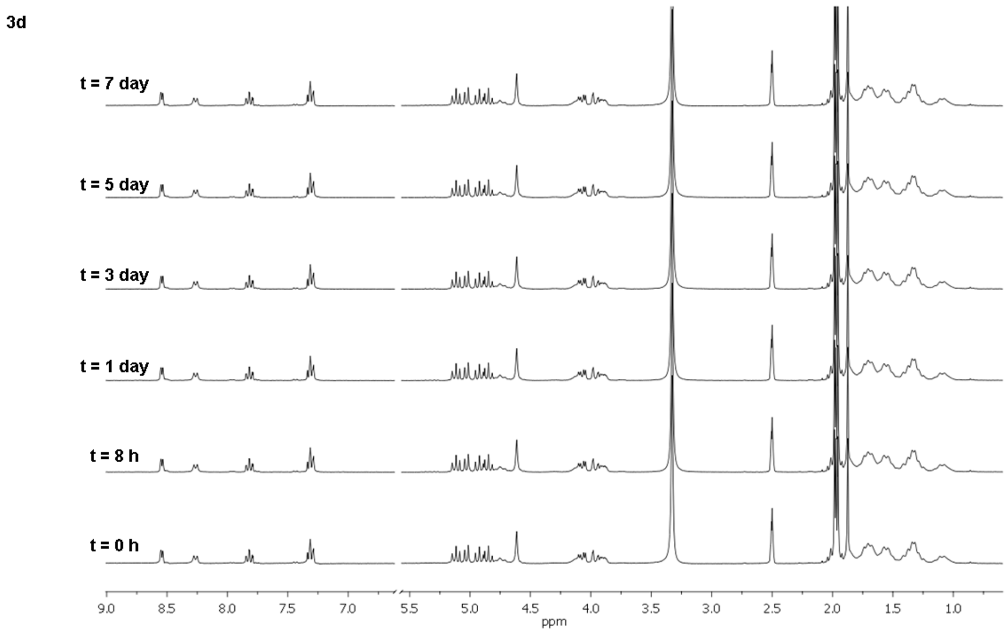

Figure S32: Stability study in ^{1}H NMR (300 MHz DMSO) of complex **3d** at different time points for 7 days.

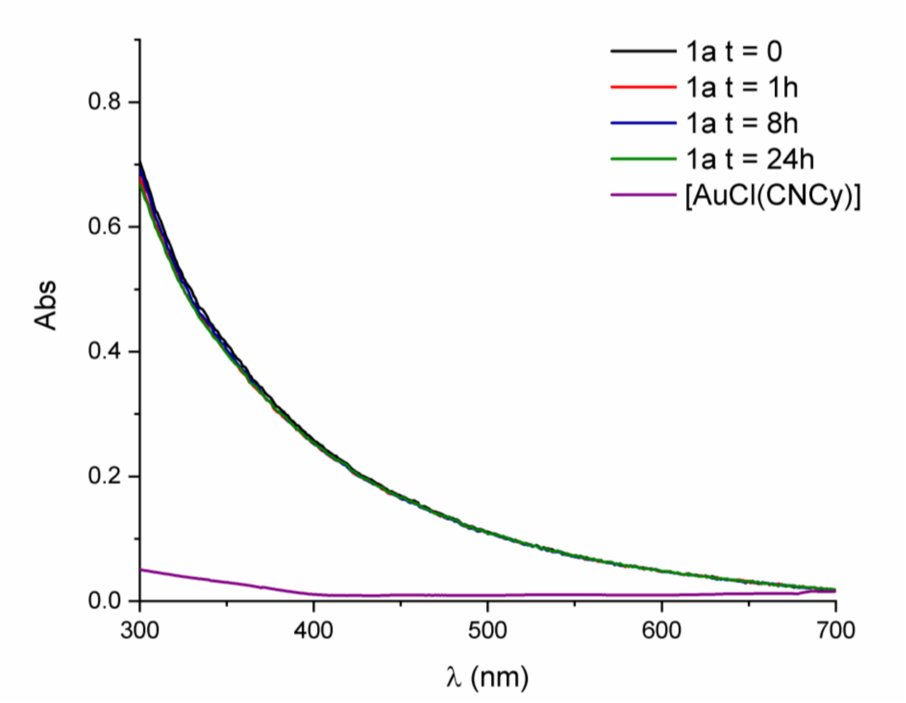

Figure S33: UV-vis spectra of complex **1a** (c = 250 μM 20% DMSO 80% PBS) at different times points and the precursor [AuCl(CNCy)] (c = 250 μM 20% DMSO 80% PBS).

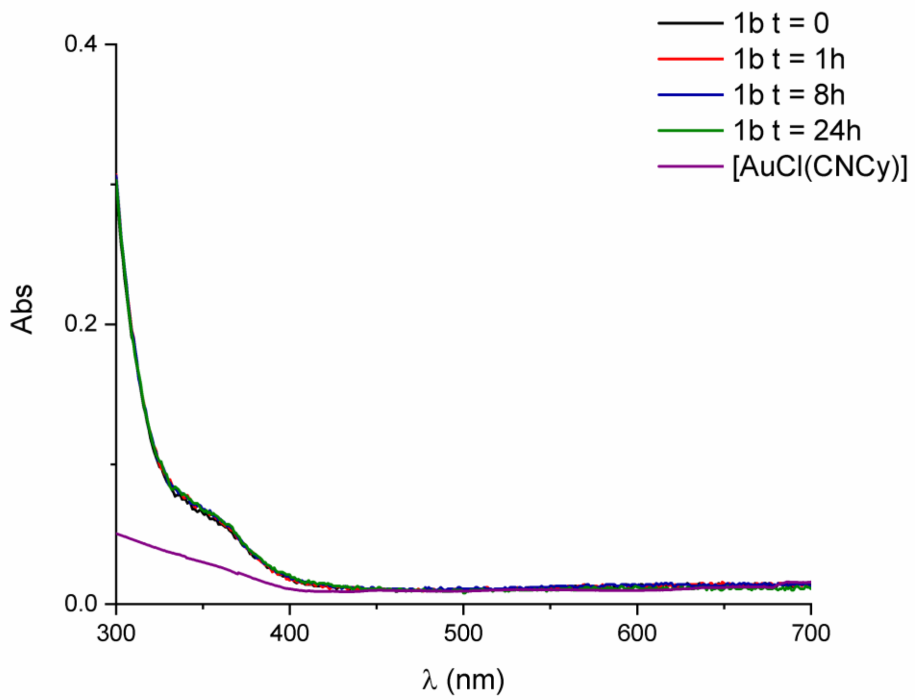

Figure S34: UV-vis spectra of complex **1b** (c = 250 µM 20% DMSO 80% PBS) at different times points and the precursor [AuCl(CNCy)] (c = 250 µM 20% DMSO 80% PBS).

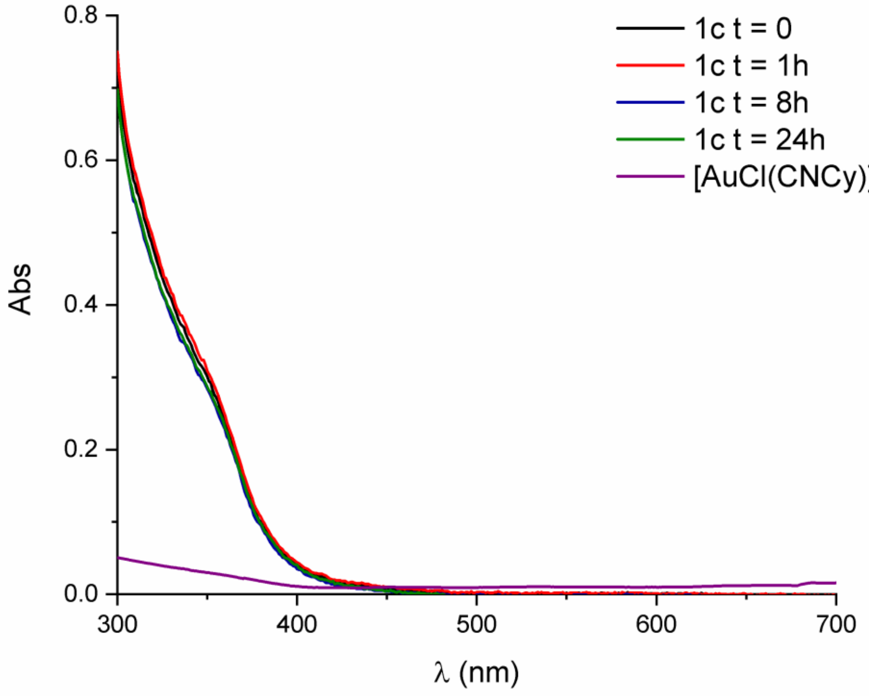

Figure S35: UV-vis spectra of complex **1c** (c = 500 μM 20% DMSO 80% PBS) at different times points and the precursor [AuCl(CNCy)] (c = 250 μM 20% DMSO 80% PBS).

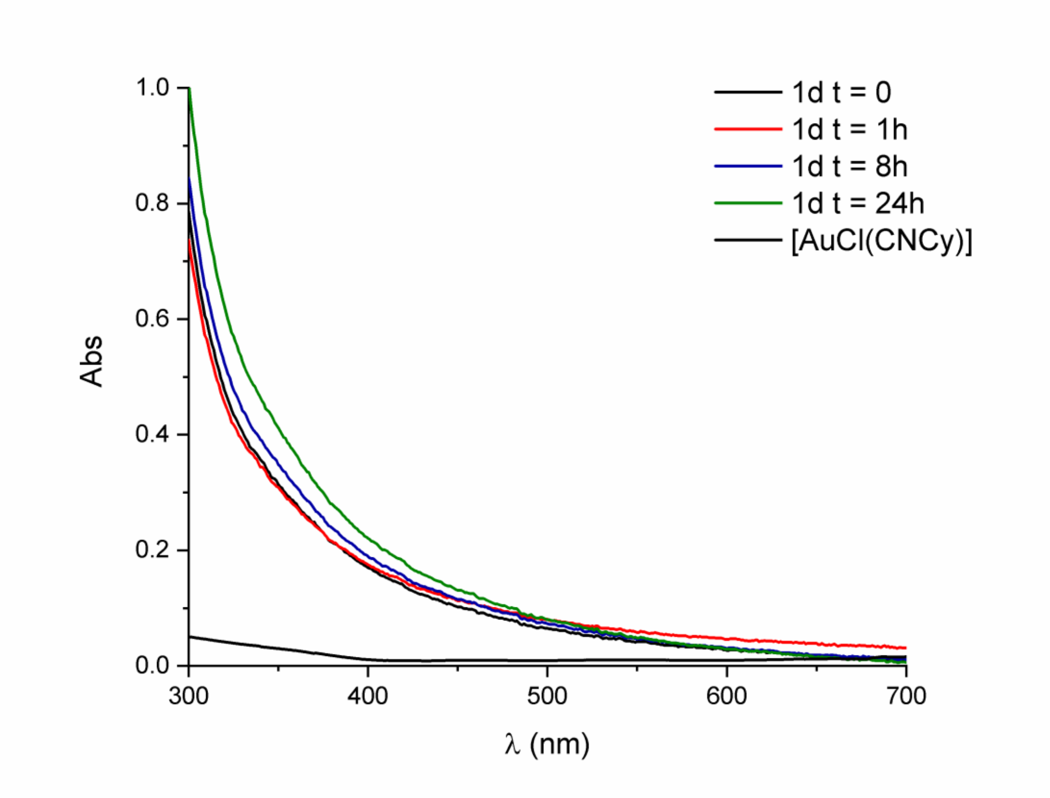

Figure S36: UV-vis spectra of complex **1d** (c = 250 μM 20% DMSO 80% PBS) at different times points and the precursor [AuCl(CNCy)] (c = 250 μM 20% DMSO 80% PBS).

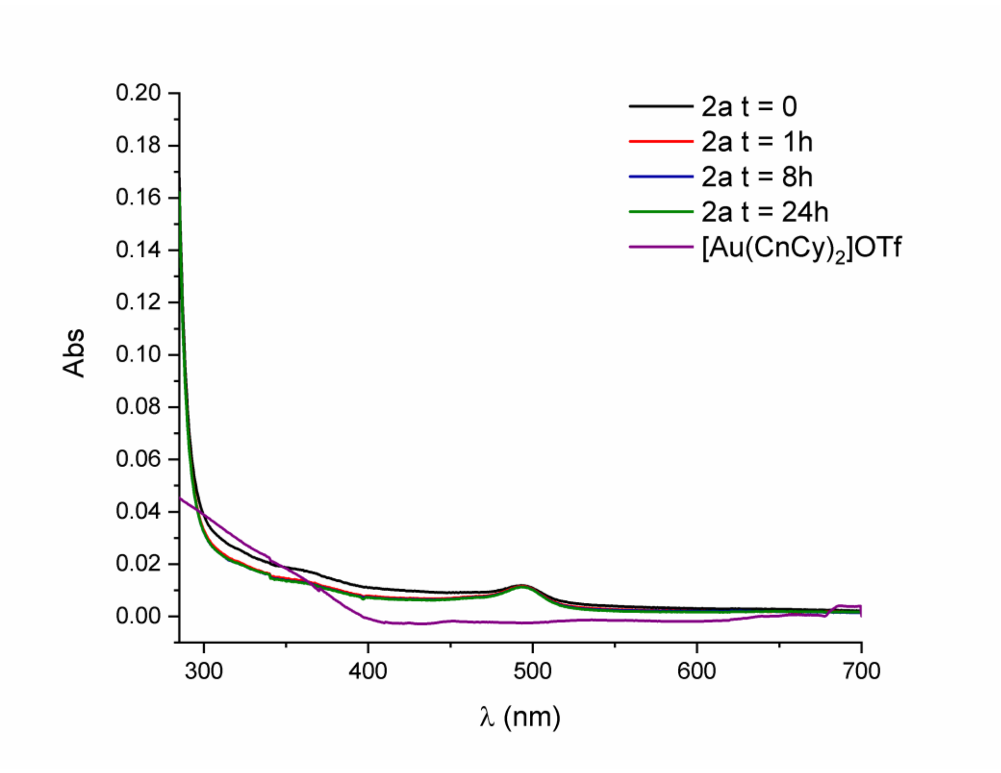

Figure S37: UV-vis spectra of complex **2a** (c = 500 μM 20% DMSO 80% PBS) at different times points and the precursor $[Au(CNCy)_2]OTf$ (c = 250 μM 20% DMSO 80% PBS).

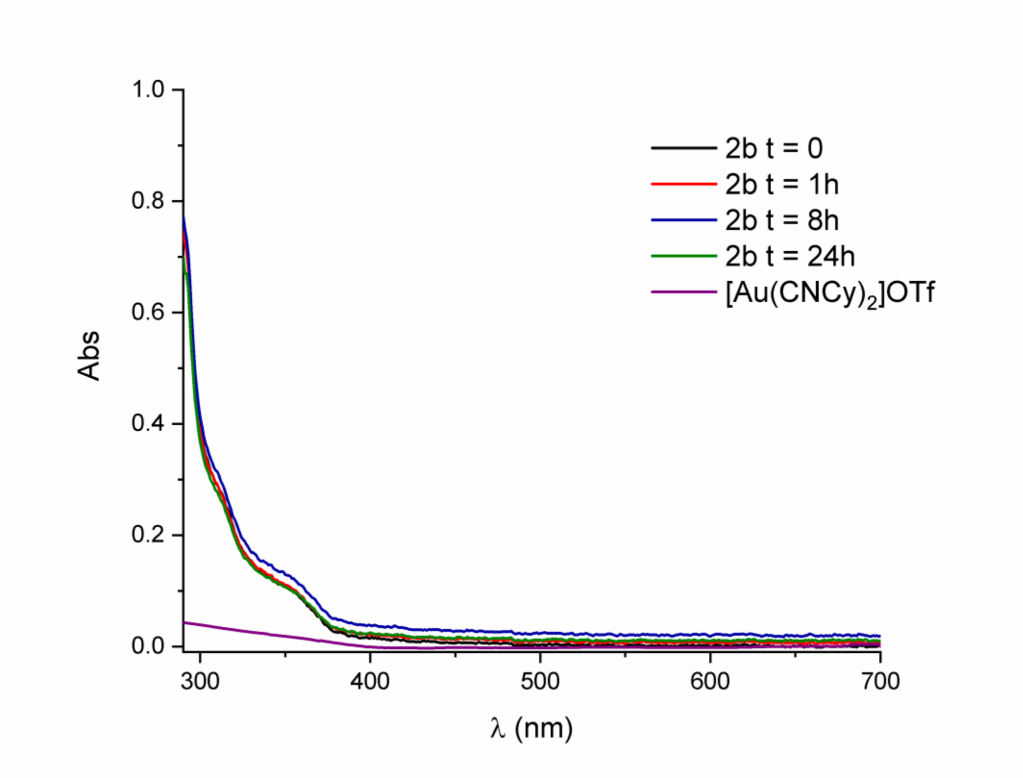

Figure S38: UV-vis spectra of complex **2b** (c = 250 μM 20% DMSO 80% PBS) at different times points and the precursor [AuCl(CNCy)] (c = 250 μM 20% DMSO 80% PBS).

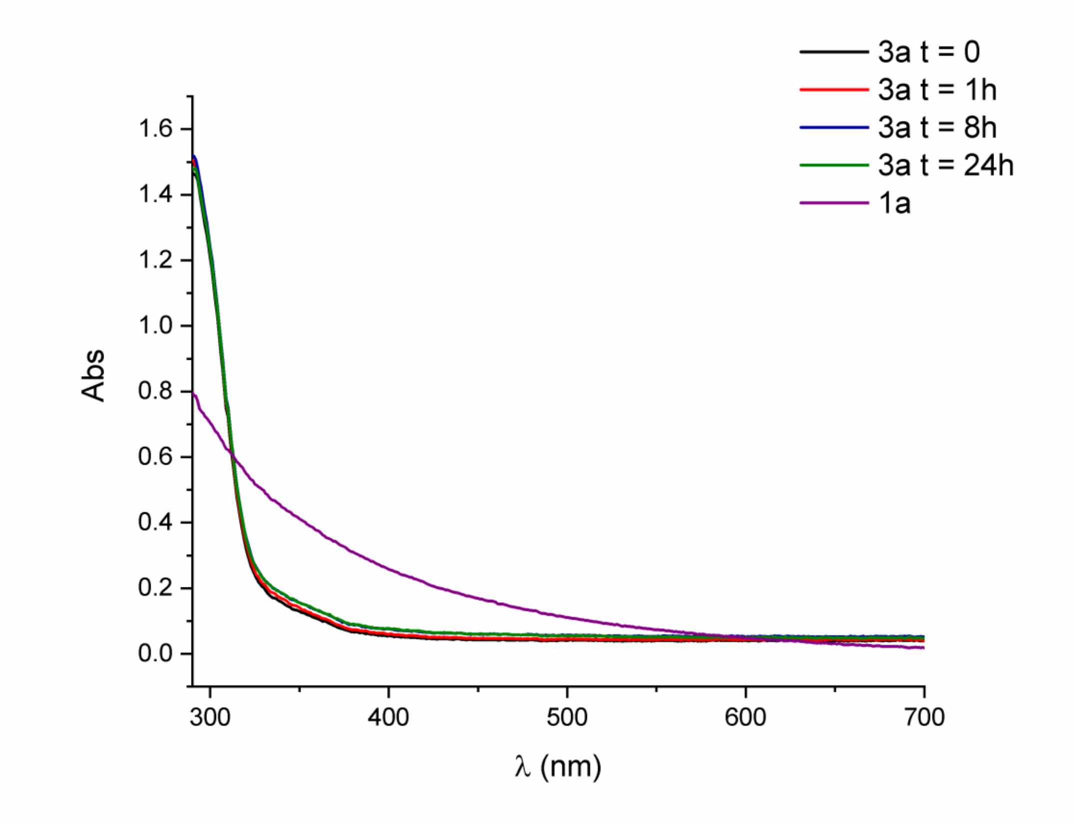

Figure S39: UV-vis spectra of complex **3a** (c = 100 µM 20% DMSO 80% PBS) at different times points and the precursor **1a** (c = 250 µM 20% DMSO 80% PBS).

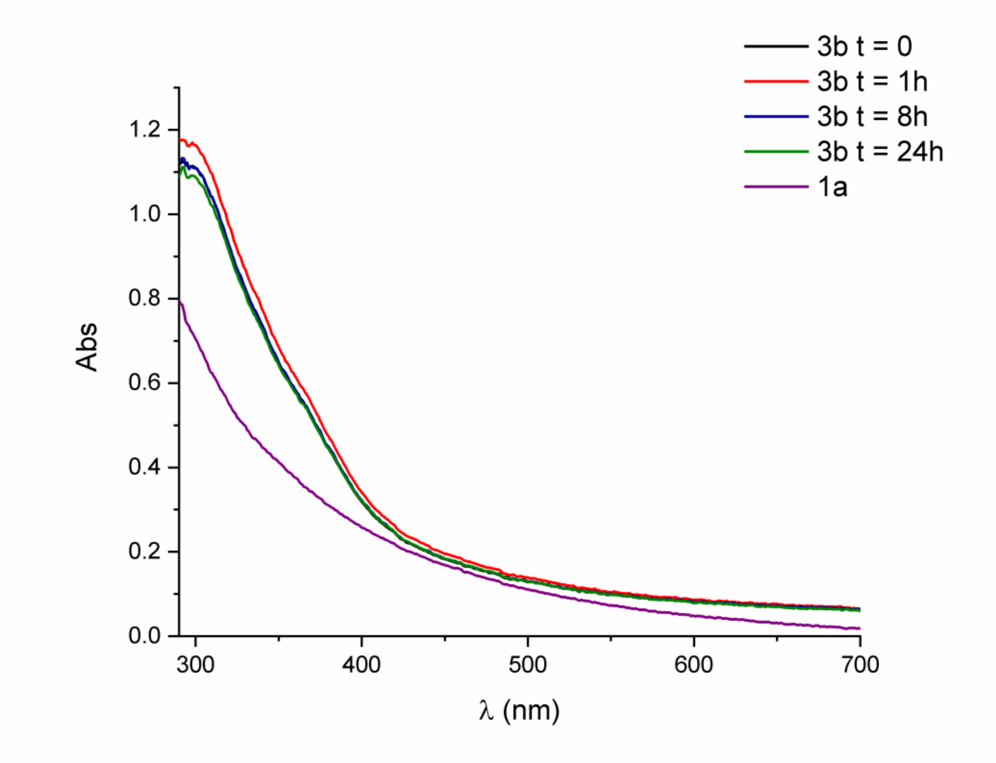

Figure S40: UV-vis spectra of complex **3b** (c = 100 µM 20% DMSO 80% PBS) at different times points and the precursor **1a** (c = 250 µM 20% DMSO 80% PBS).

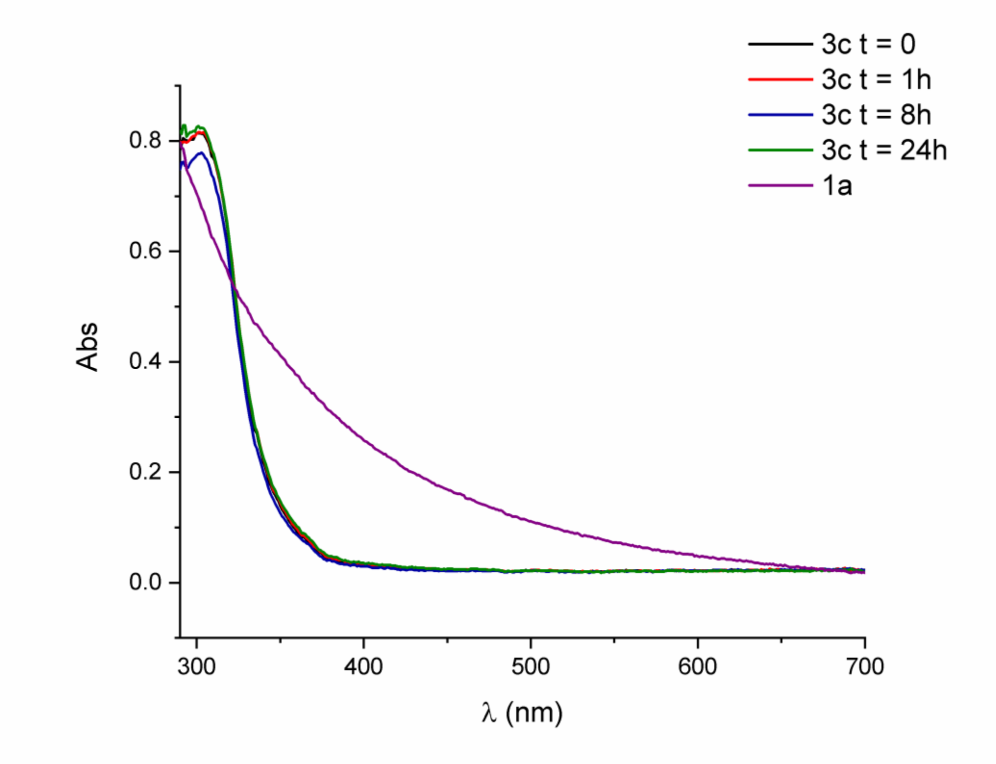

Figure S41: UV-vis spectra of complex **3c** (c = 100 µM 20% DMSO 80% PBS) at different times points and the precursor **1a** (c = 250 µM 20% DMSO 80% PBS).

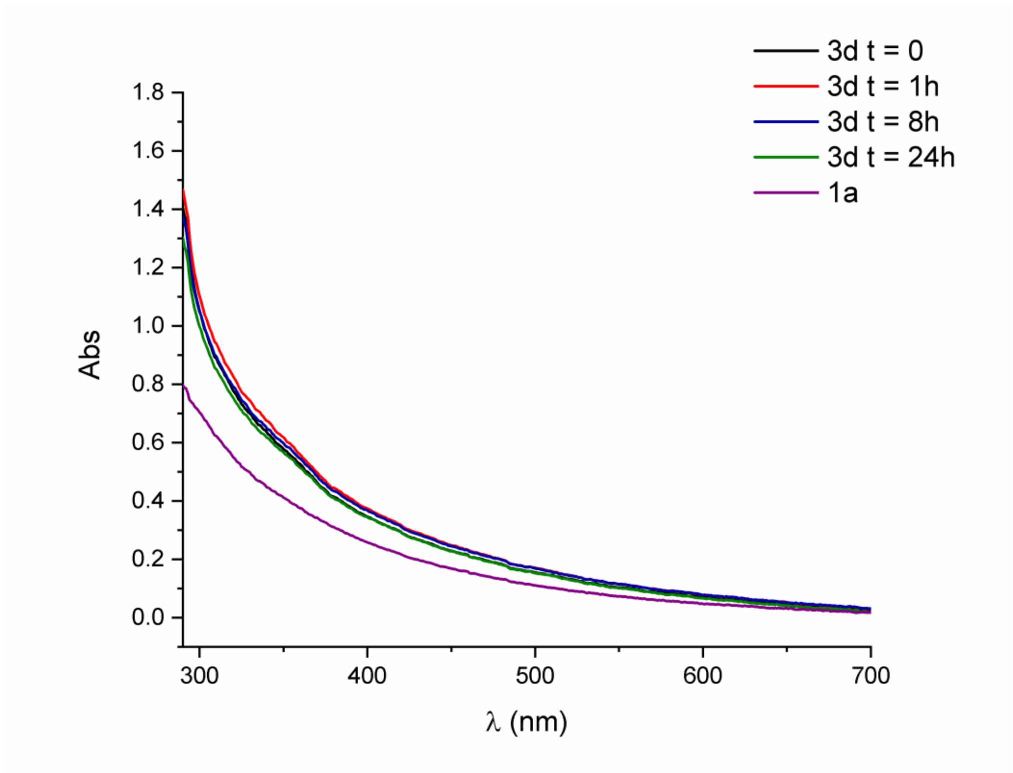

Figure S42: UV-vis spectra of complex **3d** (c = 200 μM 20% DMSO 80% PBS) at different times points and the precursor **1a** (c = 250 μM 20% DMSO 80% PBS).

4 Studies of the mechanism of cell death:

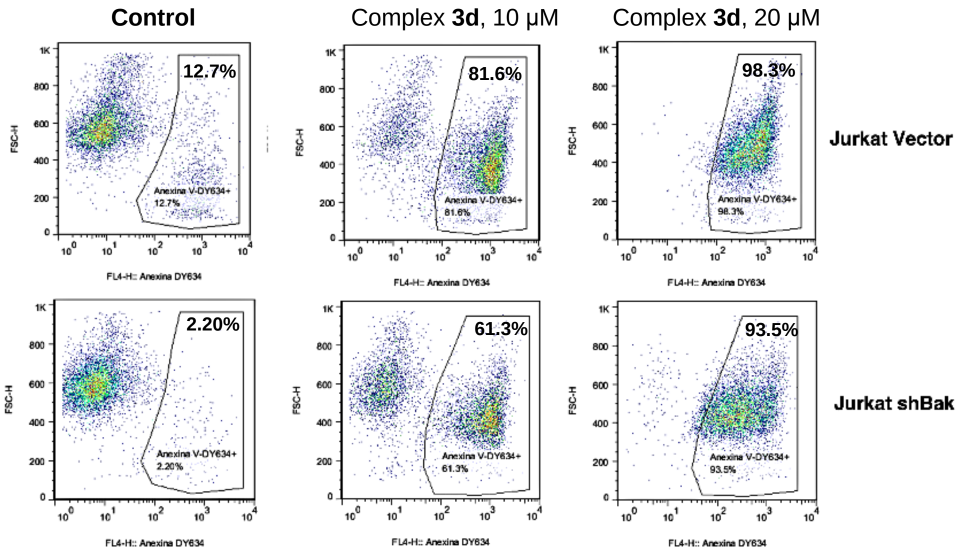

Figure S43: Flow cytometry studies on complex **3d** in Jurkat Vector and Jurkat shBak at 10 μM and 20 μM.

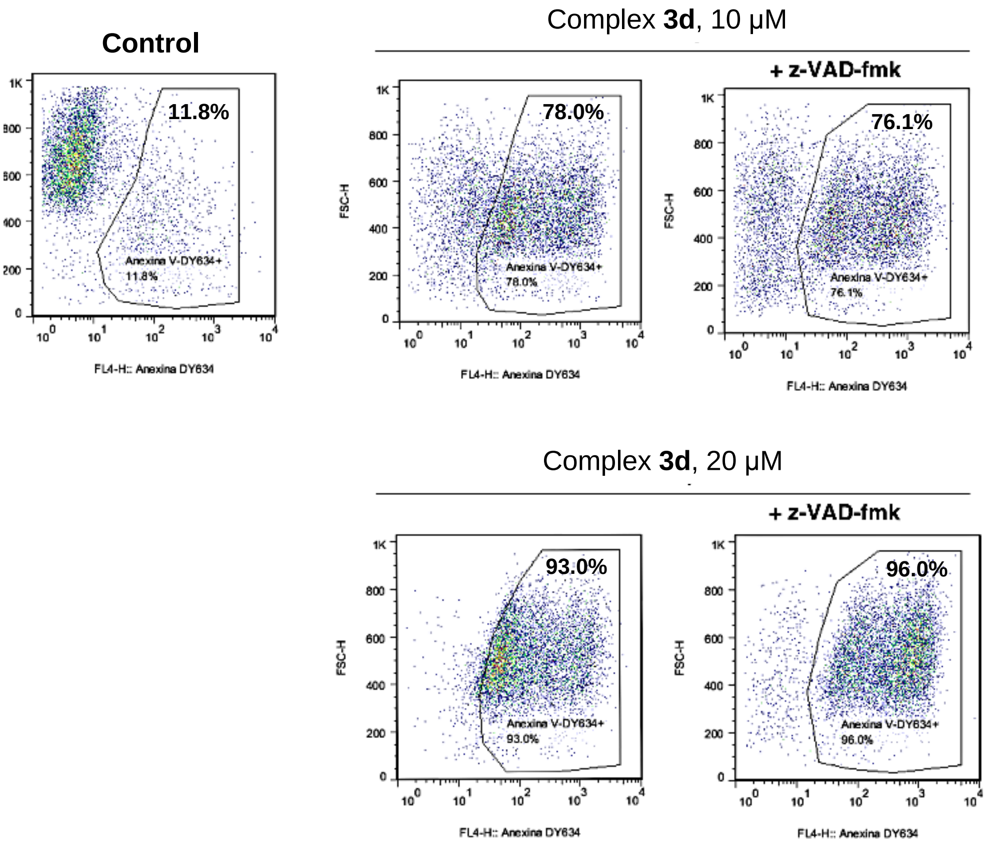

Figure S44: Flow cytometry studies on complex **3d** in Jurkat and Jurkat + z-VAD-fmk at 10 µM and 20 µM.

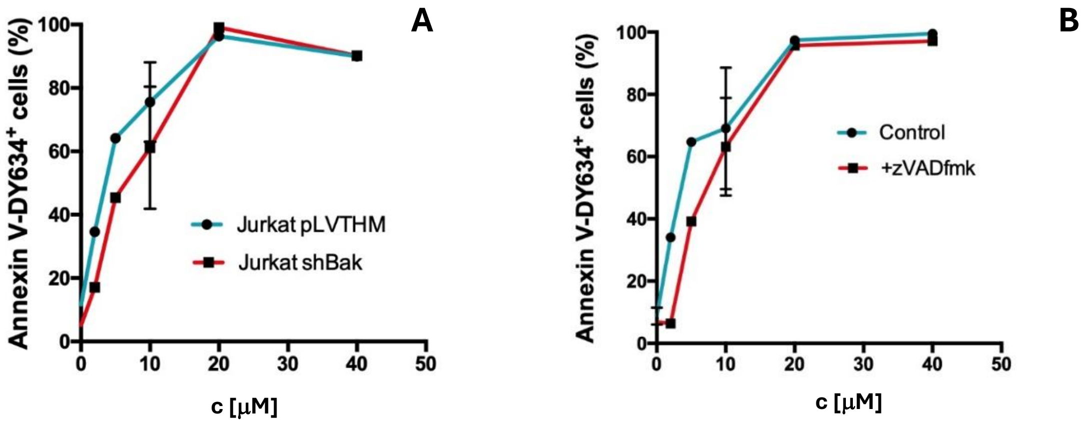

Figure S45: Graphics for the flow cytometry studies on complex **3b** at different concentrations on: a) Jurkat Vector and Jurkat shBak; b) Jurkat and Jurkat + z-VAD-fmk.

5 Flow cytometry:

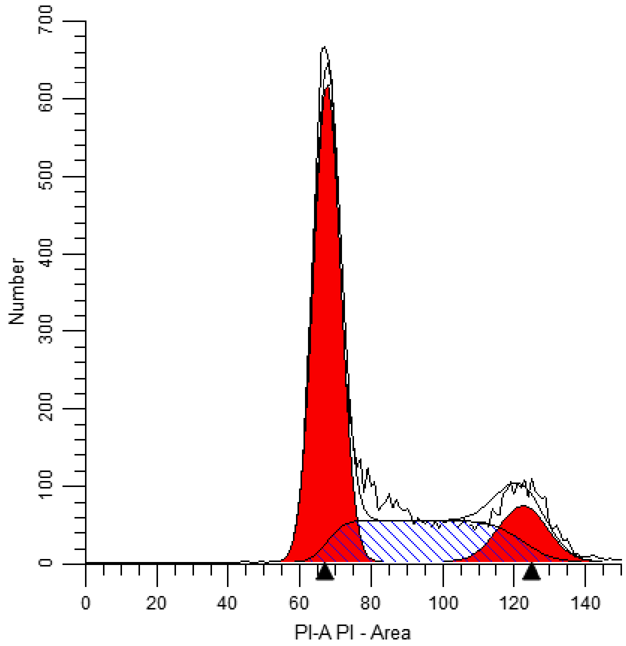

Figure S46: Cell cycle of untreated cells (control) A549

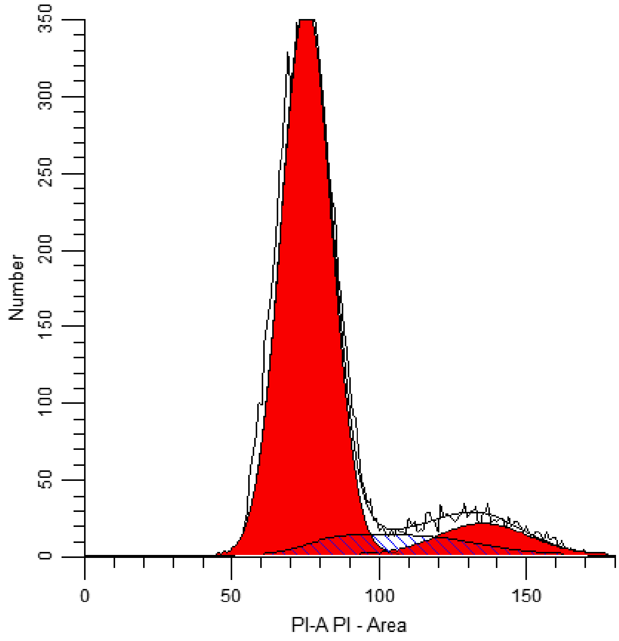

Figure S47: Cell cycle of A549 cells in presence of **3b**.

Control

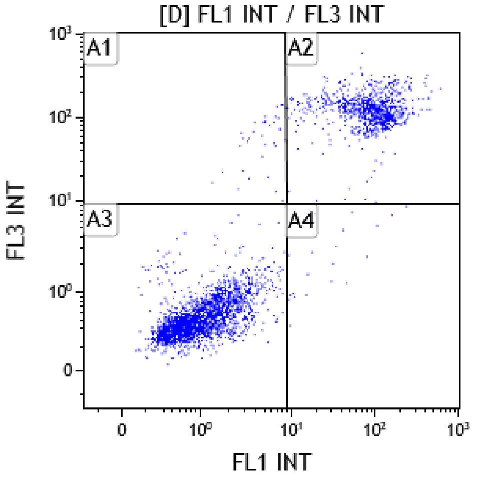

Gate	Number	%Gated
All	3.598	100,00
A1	35	0,97
A2	1.003	27,88
A3	2.539	70,57
A4	21	0,58

3b

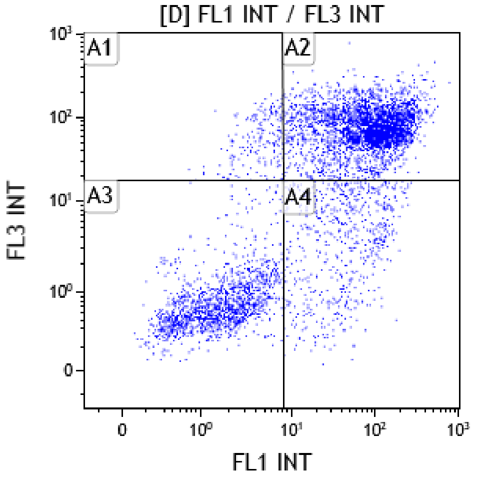

Gate	Number	%Gated
All	5.678	100,00
A1	194	3,42
A2	3.387	59,65
A3	1.461	25,73
A4	636	11,20

Figure S48: type of cell death in absence and in presence of 3b in A549 cells.
